# Diverse Signaling by TGFβ Isoforms in Response to Focal Injury is Associated with Either Retinal Regeneration or Reactive Gliosis

**DOI:** 10.1007/s10571-020-00830-5

**Published:** 2020-03-26

**Authors:** Federica Maria Conedera, Ana Maria Quintela Pousa, David Mikal Presby, Nadia Mercader, Volker Enzmann, Markus Tschopp

**Affiliations:** 1 Department of Ophthalmology, University Hospital of Bern, University of Bern, Bern, Switzerland; 2 Department of BioMedical Research, University of Bern, Bern, Switzerland; 3 Graduate School for Cellular and Biomedical Sciences, University of Bern, Bern, Switzerland; 4 Division of Endocrinology, Metabolism and Diabetes, Department of Medicine, University of Colorado Anschutz Medical Campus, Aurora, CO USA; 5 Institute of Anatomy, University of Bern, Bern, Switzerland; 6 Department of Ophthalmology, Cantonal Hospital Aarau, Aarau, Switzerland

**Keywords:** Laser injury, Tgfβ signaling, Müller cell, Retinal regeneration, Reactive gliosis, Zebrafish, Mouse

## Abstract

Müller cells may have stem cell-like capability as they regenerate photoreceptor loss upon injury in some vertebrates, but not in mammals. Indeed, mammalian Müller cells undergo major cellular and molecular changes summarized as reactive gliosis. Transforming growth factor beta (TGFβ) isoforms are multifunctional cytokines that play a central role, both in wound healing and in tissue repair. Here, we studied the role of TGFβ isoforms and their signaling pathways in response to injury induction during tissue regeneration in zebrafish and scar formation in mouse. Our transcriptome analysis showed a different activation of canonical and non-canonical signaling pathways and how they shaped the injury response. In particular, TGFβ3 promotes retinal regeneration via Smad-dependent canonical pathway upon regulation of *junb* gene family and *mycb* in zebrafish Müller cells. However, in mice, TGFβ1 and TGFβ2 evoke the p38MAPK signaling pathway. The activation of this non-canonical pathway leads to retinal gliosis. Thus, the regenerative versus reparative effect of the TGFβ pathway observed may rely on the activation of different signaling cascades. This provides one explanation of the different injury response in zebrafish and mouse retina.

**Electronic supplementary material:**

The online version of this article (10.1007/s10571-020-00830-5) contains supplementary material, which is available to authorized users.

## Background

Although the anatomical structure of the retina and its cellular composition are highly conserved across all vertebrates (Livesey and Cepko 2001), its regenerative modalities and capacities are very different. Among vertebrates, teleost can fully regenerate retinal tissue upon injury (Lenkowski and Raymond 2014). In zebrafish, the major source for endogenous retinal regeneration are Müller cells. After injury, Müller cells can rapidly dedifferentiate, proliferate, and generate progenitors that migrate to the damaged retinal layer and differentiate (Lenkowski and Raymond 2014). However, mammals are not endowed with similar ability.

Gliosis, the activation and consequent proliferation of Müller cells in response to all forms of injury and disease, is a feature of many neurodegenerative diseases of the retina (e.g., retinitis pigmentosa, glaucoma) (Bringmann and Reichenbach 2001; Bringmann et al. 2006). Müller cell reactivity has both protective and detrimental effects (Bringmann et al. 2009a). Immediately after injury, Müller cells generate neurotrophic factors to promote recovery (Garcia and Vecino 2003; Bringmann et al. 2006, 2009b). However, chronic gliosis contributes to degeneration and impedes tissue regeneration (Roche et al. 2018). Currently, the molecular and cellular requirements necessary for the successful regeneration of different organs in mammals and teleost are not well known.

Transforming growth factor β (TGFβ) signaling controls diverse cellular processes during embryogenesis as well as in mature tissues of multicellular animals. In this context, the total number of TGFβ ligands and their receptors changed only slightly in all invertebrates and jawless vertebrates. In contrast, expansion of the pathway members, especially ligands, was observed in jawed vertebrates due to the second round of whole-genome duplication (WGD) in teleosts. Thereby most receptors and their downstream targets (smads) were expressed in multiple tissues indicating they were shared by different ligands (Zheng et al. 2018). Therefore, understanding the biological role of TGFβ signaling during retinal regeneration especially in teleosts (zebrafish) may lead to identify pathways that can be leveraged for regeneration in mammals.

TGFβ signaling is essential to wound healing, including non-specific scar formation and tissue-specific regeneration (Gilbert et al. 2016). The TGFβ superfamily comprises 33 members: three multifunctional isoforms TGFβ1, TGFβ2, and TGFβ3, and downstream mediators of canonical and non-canonical signaling (Derynck and Zhang 2003). They have different and sometimes antagonistic effects on regeneration and scar formation (Casari et al. 2014). In mammals, TGFβ1 and TGFβ2 promote collagen deposition and scar formation, while TGFβ3 is anti-fibrotic (Ferguson et al. 2009). In zebrafish, the TGFβ pathway is involved in regenerating heart, fin and retina (Chablais and Jazwinska 2012; Jazwinska et al. 2007; Wan and Goldman 2016). In a light-induced model of retinal injury in zebrafish, TGFβ1 is initially upregulated but then subsequently suppressed during the proliferative, neurogenic Müller cell response (Lenkowski et al. 2013). In a chemical-induced model of retinal injury in zebrafish, blocking TGFβ signaling with SB431542 leads to increased Müller cell proliferation (Tappeiner et al. 2016).

Here, we examined gene and protein levels of the TGFβ family members after injury, specifically focusing on TGFβ1, TGFβ2, and TGFβ3 isoforms. We also analyzed key downstream signaling mediators that are associated with tissue regeneration in zebrafish and scar formation in mouse. Comparing zebrafish and murine Müller cell transcriptome, we observe that canonical and non-canonical TGFβ signaling is activated differently and these pathways may distinctly contribute to either a reparative or restorative response after injury.

## Materials and Methods

### Animals

Experiments were performed in zebrafish (license n. BE34/19) and mice (license n. BE33/18). Both were approved by the ethics committee for involving animals in research of the Canton of Bern (Switzerland).

Transgenic TgBAC (gfap:gfap-GFP) zebrafish (AB strain; European zebrafish Resource Center, Karlsruhe, Germany) have been described previously (Rao et al. 2017). Only adult zebrafish (> 5.5 month of age) were used in this study. They were kept under standard conditions in tank water with a temperature of approximately 26.5 °C and raised in a 14/10 h light/dark cycle (Avdesh et al. 2012; Sprague et al. 2008). They were fed dry food twice per day (GEMMA Micro 300; Westbrook, ME, USA and TetraMin Tropical Flakes; Delphin-Amazonia AG, Münchenstein, Switzerland) and *Artemia salina* once per day. During experiments, animals were kept in tank water. Male and female zebrafish were randomly selected to be treated with 20 mg/l tranexamic acid (TXA; OrPha Swiss GmbH, Küsnacht, Switzerland) dissolved in tank water. Zebrafish were immersed 12 h before the induction of the retinal injury and maintained under those conditions for a maximum of 14 days. The TXA treatment was renewed every day. Animals were observed daily during the treatment period for any changes in behavior and those showing substantial weight loss, morphological changes or swimming behavioral alterations were excluded from the study. Additionally, we used zebrafish embryo staged at 48–72 h post fertilization as a positive control for testing the antibodies.

Rlbp1-GFP mice express GFP under control of the retinaldehyde binding protein 1 (Rlbp1) promoter in Müller cells as described before (Vazquez-Chona et al. 2009). During experimentation, mice were housed in groups of 2–5 under temperature and humidity-controlled conditions in individually ventilated cages with a 12 h light/dark cycle with food and water available ad libitum. Mice were genotyped by PCR amplification of genomic DNA from ear biopsies and the following conventional PCR conditions: initial denaturation (94 °C, 5 min); 30 cycles of denaturation (94 °C, 30 s), annealing (61 °C, 1 min) and elongation (72 °C, 30 s); final extension (72 °C, 10 min). The following primers (Microsynth, Balgach, Switzerland) were used: Rlbp1-GFP (5′-CAAGTGTGAGAGACAGCATTGC-3′, reverse 5′-GTCGGCCATGATATAGACGTTG-3′). PCR products were run on a 1.4% agarose gel with 1% TBE buffer for size detection.

### Retinal Laser Focal Injury

In zebrafish, after anesthesia with 0.16 mg/mL ethyl 3-aminobenzoate methanesulfonate salt (Tricaine; Sigma-Aldrich, Buchs, Switzerland) dissolved in the tank water, 1–2 drops of 2% hydroxypropyl methylcellulose (Methocel, OmniVision AG, Neuhausen, Switzerland) were topically applied to the cornea. A diode laser with a wavelength of 532 nm (Visulas 532 s, Carl Zeiss Meditec AG, Oberkochen, Germany) was used to create retinal lesions at the region of the posterior pole around the optic nerve (Conedera et al. 2017). These were confined to the ONL (outer nuclear layer) and surrounded by healthy tissue. Each burn was produced with 70 mW of power for 100 ms and aimed to have a diameter of 50 µm (Conedera et al. 2017). The right eye was used as internal negative control. To focus, the laser-aiming beam on the retina a 2.0 mm laser lens was employed (Ocular Instruments, Bellevue, WA, USA). After laser damage, zebrafish were revived by placing them in a container with fresh tank water and creating a water flow over the gills. In the murine model, anesthesia was performed subcutaneously by injecting 45 mg/kg ketamine (Ketalar 50 mg/mL; Orion Pharma AG, Zug, Zurich, Switzerland) and 0.75 mg/kg medetomidine hydrochloride (Domitor, 1 mg/mL; Orion Pharma AG). Pupils were dilated using tropicamide 0.5% and phenylephrine HCl 2.5% (ISPI, Bern, Switzerland). Afterwards, few drops of 2% hydroxypropyl methylcellulose were applied topically to the cornea before the treatment. The same diode laser employed for the zebrafish was used in mouse to damage the ONL. Each burn was 100 µm in diameter and produced with 120 mW of power for 60 ms. The right eye was used as internal negative control. After laser damage induction, 0.75 mg/kg atipamezole (Antisedan 5 mg/mL; Orion Pharma AG) was given to reverse the anesthesia. Additionally, 200 μL of 0.9% NaCl was injected subcutaneously to prevent dehydration.

For in vivo and histological analysis, zebrafish received four laser burns to the left eye: two superior and two inferior to the optic nerve separated from the nerve by at least two lesion diameters. In mouse, six laser burns were created in the left eye: three superior and three inferior to the optic nerve separated from the nerve by at least two lesion diameters. For the RNA-seq analysis, 20 and 50 laser burns were induced in both eyes in the zebrafish and mouse retinas, accordingly. We generated as many laser burns as we could, maintaining a space of at least one spot size between each laser burn.

The difference in laser burn numbers is due to the different size of the fish and murine eyes. This limits our ability to induce the same number of laser burns to damage the fundus of the eye. However, we did not notice tremendous differences in the outcome since the injury itself was induced with the same aim (damage the photoreceptor layer) and, roughly, the same density of laser burns.

### In Vivo Imaging

Zebrafish were placed in 0.16 mg/mL tricaine solution until they became immobile and did not respond to external stimuli. Each zebrafish was transferred to a custom-made silicone pin holder for imaging. To obtain optimal images, we adapted a commercially available hydrogel contact lens (Johnson & Johnson AG, Zug, Switzerland) to fit the zebrafish eye (*Ø* = 5.2 mm, *r* = 2.70 mm, center thickness = 0.4 mm). The concave surface of the lens was placed over the cornea using Methocel (OmniVision AG).

Mice were anesthetized as described above and placed on a custom-made platform positioned on the chin rest of the spectral domain—optical coherence tomography (SD-OCT) device. No contact lens was used during image acquisition. Pupils were dilated with a drop of tropicamide 0.5% phenylephrine 2.5% (ISPI), and methocel (OmniVision AG) applied to each eye during imaging to keep the cornea hydrated.

Standard confocal laser scanning ophthalmoscope (Heidelberg Spectralis HRA + OCT; Heidelberg Engineering GmbH, Heidelberg, Germany) equipped with 78D non-contact slit lamp lens (Volk Optical, Mentor, OH, USA) was used to image both animal models (DiCicco et al. 2014; Liu et al. 2013). The infrared (IR) mode was used to focus on retinal vessels at high resolution of 1536 × 1636 pixels. After examination of both eyes, SD-OCT was performed using a 55° lens at a high resolution of 1008 × 596 pixels in grid mode. In total, 25 to 50 images were acquired centered on the optic nerve head. Representative examples were selected for the figures. The bold green lines show the location of the OCT images in the retina.

After imaging, zebrafish were revived in tank water. However, for mice, atipamezole (2.3 mg/kg, Antisedan 5 mg/mL, Provet AG, Lyssach, Switzerland) was used to antagonize medetomidine and awake them.

### Tissue Processing and Histology

Zebrafish were euthanized by submersion in ice-cold (4 °C) anesthesia solution and mice were euthanized by carbon dioxide. The eyes were enucleated at designated times [1, 3, 7 and 14 days post laser induction (dpli)] after damage induction and fixed with 4% paraformaldehyde (PFA) in phosphate-buffered saline (PBS) in both animal models overnight. Afterward, the eyes were embedded in paraffin and 5 μm sections were sliced as previously described (Tappeiner et al. 2013). The sections were stained with Mayer’s hemalum and eosin (H&E; Roth, Karlsruhe, Germany) (Conedera et al. 2017).

### Image Analyses and Quantification of Histological Sections

The area of each lesion detected by non-invasive imaging technique (OCT) was measured by determining the length of the hyper-reflective signal in both animal models using the Heidelberg Eye Explorer software (Heidelberg Engineering GmbH). High-throughput and high-quality brightfield H&E-stained images of the ONL at 40 × total magnification were acquired with a motorized Pannoramic 250 Flash II microscope (3DHISTECH Ltd., Budapest, Hungary). Sagittally oriented retinal sections at the level of the laser burn were used. The analyzed length of the retina was 50 or 100 µm, corresponding to the induced laser burn size in zebrafish and mouse, respectively. The ONL nuclei were outlined manually and bucket-filled-in GNU Image Manipulation Program (GIMP 2.10.8). Images were analyzed in ImageJ v1.39 (Wayne Rasband; NIH, Bethesda, MD, United States).

### Quantitative Real-Time PCR

For qRT-PCR analysis, we isolated fish and murine retinas at different time points (1, 3, 7 and 14 dpli) after injury (20 laser burns for zebrafish and 50 laser burns for mouse) and in negative controls (uninjured retinas from age-matched, undamaged siblings). Total RNA was isolated using the RNeasy Micro Kit (Qiagen, Hombrechtikon, Switzerland) according to the manufacturer’s instructions. Three independent samples obtained from four pooled retinas were used for each condition. The cDNA was reverse transcribed by the iScript cDNA Synthesis Kit (Bio-Rad, Cressier, Switzerland) according to the manufacturer’s instructions and quantified using a NANODROP 1000 spectrophotometer (ThermoScientific, Basel, Switzerland). The gene-specific primers used for the zebrafish: *tgfβ1a* forward 5′- GAAGGCAACACAAGGTGGAG-3′ and reverse 5′- CCCGACTGAGAAATCGAGCC-3′; *tgfβ2* forward 5′-GAGACGCGCTTTGCAGGTAT-3′ and reverse 5′-GCTCTTATGCTGCGACTCCA-3′; *tgfβ3* forward 5′-CCGCTCAGATATGGGTCGTC-3′ and reverse 5′-CGCAGCAGTTCTCCTCGTAA-3′ and *gapdh* forward 5′-ATGACCCCTCCAGCATGA-3′ and reverse 5′-GCGGTGTAGGCATGAAC-3′. The following primer pairs were used for the mouse model: *Tgfβ1* forward 5′-AGCTGCGCTTGCAGAGATTA-3′ and reverse 5′-AGCCCTGTATTCCGTCTCC-3′; *Tgfβ2* forward 5′-TCCCCTCCGAAAATGCCATC-3′ and reverse 5′-ACTCTGCCTTCACCAGATTCG-3; *Tgfβ3* forward 5′- ATGACCCACGTCCCCTATCA-3′ and reverse 5′- AGTTCATTGTGCTCCGCCAG-3′ and *Gapdh* forward 5′- AACTTTGGCATTGTGGAAGG-3′ and reverse 5′- ACACATTGGGGGTAGGAACA-3′. qRT-PCR was performed using the CFX Connect™ Real-Time PCR Detection System (Bio-Rad). Cycle thresholds were normalized against the reference gene (Gapdh). Expression data are presented as means ± SD calculated against the negative control samples. Expression in control samples was set to ‘1’.

### Immunofluorescence

Paraffin tissue sections were also used for immunofluorescence analyses. Sections were boiled in Tris–EDTA (pH 9.0) or Citrate buffer (pH 6.0) with 0.05% Tween-20 for 4 min and then cooled at room temperature (over ~ 30 min). All retinal sections were blocked for 1 h in Tris-buffered saline (TBS; pH 7.6) + 10% goat normal serum (DAKO, Agilent Technologies, Baar, Switzerland) + 1% bovine serum albumin (Sigma-Aldrich) and incubated with primary antibodies overnight at 4 °C. Primary antibodies used in this study were mouse anti-glutamine synthetase (GS; 1:200; MAB302; Millipore, Billerica, MA, USA), rabbit anti-GS (1:200; ab210107; Abcam), rabbit anti-transforming growth factor beta 1 (Tgfβ1; 1:200 dilution; ab215715; Abcam, Cambridge, UK), mouse anti-transforming growth factor beta 2 (Tgfβ2; 1:50 dilution; ab36495; Abcam), rabbit anti-transforming growth factor beta 3 (Tgfβ3; 1:100 dilution; ab15537; Abcam), rabbit anti-p38 mitogen-activated protein kinase (p38MAPK; 1:500 dilution; 4511; Cell Signaling Technology, Danvers, MA, USA); rabbit anti-plasminogen activator inhibitor type 1 (PAI1; 1:200 dilution; ab226946; Abcam) and rabbit anti-connective tissue growth factor (CTGF; 1:200 dilution; ab6992; Abcam). As secondary antibodies, goat anti-rabbit/anti-mouse Alexa 488 nm/594 nm (1∶500; Life Technologies, Paisley, UK) diluted in TBS with 1% BSA were used for 1 h at room temperature. The cell nuclei were counterstained using Vectashield with 4′, 6-diamidino-2-phenylindole (DAPI; Vector Labs, Burlingame, CA, USA). All antibodies used for immunofluorescence in zebrafish were validated in embryo sections (positive controls).

Image analyses and quantification of immunofluorescence sections. Imaging was performed at 40 × total magnification with a scanning laser microscope (Zeiss LSM710; Carl Zeiss Microscopy, Jena, Germany). Sagittally oriented retinal sections at the level of the laser burn were used to quantify positive cells. The analyzed length of the retina was 50 or 100 µm, corresponding to the size of the induced laser burn, both in zebrafish and mouse models, respectively. Therein, the number of positive cells in the INL and the ONL was manually determined. Afterwards the number of the positive cells was normalized to the total number of cells (GS^+^). Ratios were expressed as percentages.

### Flow Cytometry Analysis

At different time points after laser induction (1 and 3 dpli), retinas of Rlbp1:GFP mice were used for flow cytometry analysis. Both retinas of each mouse were analyzed as one sample. Retinas were processed according to Ebneter et al. (Kokona et al. 2018). Before antibody staining, single cells suspensions were incubated with Hoechst 33342 Ready Flow™ Reagent (ThermoFisher Scientific) in Hank's Balanced Salt Solution (HBSS; ThermoFisher Scientific) with DNase I (200 U/mL; Roche), for cell death detection, according to the manufacturer’s instructions. For antibody staining, the samples were washed and re-suspended in flow cytometry buffer (HBSS with 20% FBS and 200 U/mL DNase I). Activated Müller cells and reactive oxygen species (ROS) production were subsequently stained with fluorescent-labeled antibodies against glial fibrillary acid protein (GFAP; Alexa Fluor® 488 anti-GFAP antibody, 2E1.E9; Biolegend, San Diego, CA, USA) and with CellROX Deep Red oxidative stress reagent (5 μM; ThermoScientific) at 4 °C in the dark for 30 min, respectively. Samples were washed again and re-suspended in 0.1% PFA (pH 7.4) at 4 °C in the dark for 10 min. Samples were washed twice, re-suspended in flow cytometry buffer and then analyzed. All washing steps involved addition of 1 mL HBSS with 0.01% DNase on each sample and centrifugation at 300×*g* at 4 °C for 3 min. Data were acquired with an LSR II Cytometer System and the BD FACSDiva software (BD Biosciences, Allschwil, Switzerland). The data were analyzed with the Flowjo Single Cell Analysis Software V10 (TreeStar, Ashland, OR, USA).

### Retinal Dissociation, Sorting, and RNA-Seq Library Production

At different time points after injury induction (1, 3, and 7 dpli), both retinas of three gfap:gfap-GFP zebrafish per time point were dissected and kept in cold diethyl pyrocarbonate phosphate-buffered saline (DEPC-PBS) (Glaviano et al. 2016). Enzymatic dissociation was initiated by 0.05% trypsin (ThermoFisher Scientific) diluted in DEPC-PBS at 37 °C for 10 min. Cell suspension was then mechanically triturated, trypsin inhibitor (10 mg/mL; Sigma-Aldrich) added and centrifuged at 1200 rpm for 5 min. Dissociated retinas were re-suspended in DEPC-PBS with 10% FBS and DNase I (200 U/mL; Roche), filtered and collected in Falcon® Round-Bottom Tubes with CellStrainer Cap (12 × 75 mm; Costar Corning, Cambridge, MA, USA). Hoechst 33342 Ready Flow™ Reagent (ThermoFisher Scientific) was added as a DNA dye for cell cycle analysis. Cells from gfap:gfap-GFP negative siblings were used to determine background fluorescence levels. 100 cells/μL above this threshold were collected from gfap:gfap-GFP positive zebrafish using fluorescence-activated cell sorting (FACS) into separate 96-well plates containing 4 μL lysis buffer comprised of Buffer TCL (1031576; Qiagen) plus 1% 2-mercaptoethanol (63689; Sigma-Aldrich).

For RNA-seq experiments using the Rlbp1:GFP mice, retinas were collected in HBSS at different time points (1, 3, and 7 dpli) and promptly dissociated as described (Brady and Iscove 1993; Dulac and Axel 1995; Tietjen et al. 2003; Trimarchi et al. 2007). Briefly, both left and right retinas from an adult mice (in triplicate) were dissected and incubated with papain (Worthington Biochemical, Freehold, NJ, USA) for 10–15 min at 37 °C. Papain was removed with one wash in HBSS with 10% FBS and DNase I (200 U/mL; Roche), after which retinas were placed in HBSS containing 0.4% BSA (A8806; Sigma-Aldrich) and DNase I (200 U/mL; Roche), dissociated by trituration, passed through a 35 μm cell strainer, and placed on ice. Hoechst 33342 Ready Flow™ Reagent (ThermoFisher Scientific) was added as a DNA dye for cell cycle analysis. Cells from Rlbp1:GFP negative littermates were used to determine background fluorescence levels. 100 cells/μL above this threshold were collected from Rlbp1:GFP positive animals using FACS into separate 96-well plates with 4 μL lysis buffer comprised of Buffer TCL (1031576; Qiagen) plus 1% 2-mercaptoethanol (63689; Sigma-Aldrich).

After cell sorting, all samples were immediately frozen at − 70 °C. Sorted cells were processed using the published Smart-seq2 protocol (Picelli et al. 2014) to generate the cDNA libraries. The quality of the RNA, cDNA and final libraries was determined using an Agilent 4200 TapeStation System (Agilent, Santa Clara, CA, USA). The libraries were pooled and sequenced in an Illumina HiSeq4000 (Illumina, San Diego, CA, USA) with a depth of around 20 Mio reads per sample.

### RNA-Seq Analysis

The raw reads were first cleaned by removing adapter sequences, trimming low quality ends, and filtering reads with low quality (phred quality < 20) using Trimmomatic (Version 0.36). The read alignment was done with STAR (v2.6.0c) (Dobin et al. 2013). As reference we used the Ensembl zebrafish genome build GRCz10 from 2017-06-07 (release 89) and respectively the Ensembl mouse genome build GRCm38.p5 with the gene annotations downloaded on 2018-02-26 from Ensembl (release 91). The STAR alignment options were"—outFilterType BySJout—outFilterMatchNmin 30—outFilterMismatchNmax 10—outFilterMismatchNoverLmax 0.05—alignSJDBoverhangMin 1—alignSJoverhangMin 8—alignIntronMax 1000000—alignMatesGapMax 1000000—outFilterMultimapNmax 50". Gene expression values were computed with the function “featureCounts” from the R package “Rsubread” (v1.26.0) (Li and Dewey 2011). The options for “featureCounts” were: min mapping quality 10—min feature overlap 10 bp—count multi-mapping reads—count only primary alignments—count reads also if they overlap multiple genes. To detect differentially expressed genes we applied a count based negative binomial model implemented in the software package DESeq2 (R version: 3.5.0, DESeq2 version: 1.20.0). The differential expression was assessed using an exact test adapted for over-dispersed data. Genes showing altered expression with an adjusted *p* value < 0.05 (Benjamini and Hochberg method) were considered differentially expressed.

Heatmaps were generated for selected subsets of genes in R v. 3.5.1 using the heatmap.2 function from package gplots (v. 3.0.1.). The data displayed the log2 fold-changes between two experimental groups. Rows are reordered based on a dendrogram from hierarchical clustering.

To generate principle component analysis (PCA), data were first cleaned to remove any genes that had expression values of 0 in 10 out of 12 of the mouse or zebrafish data sets. Principle components were then calculated in R using the base function “prcomp,” with the center and scale argument set to "TRUE." Data were graphed using the “ggbiplot” package with the obs.scale set to 1 and ellipses were drawn around groups using the inherent ellipse argument.

### Statistical Analysis

Statistical analysis was performed using GraphPad Prism (version 7.0, GraphPad Software, La Jolla, USA). Intergroup comparisons were based on a non-parametric one-/two-way analysis of variance (ANOVA) and the Bonferroni multiple comparison post hoc test. For the TXA-treated zebrafish, comparison between uninjured and 14 dpli was performed with two-tailed t test. Quantifications were done on three laser burns performed in the left eye in four different animals for all time points (*n* = 12). All results are expressed as the mean ± standard deviation (SD). The level for statistical significance was set at a *p* value ≤ 0.05.

## Results

### Kinetics of Retinal Degeneration and Regeneration After Damage Induction

**Fig. 1:**
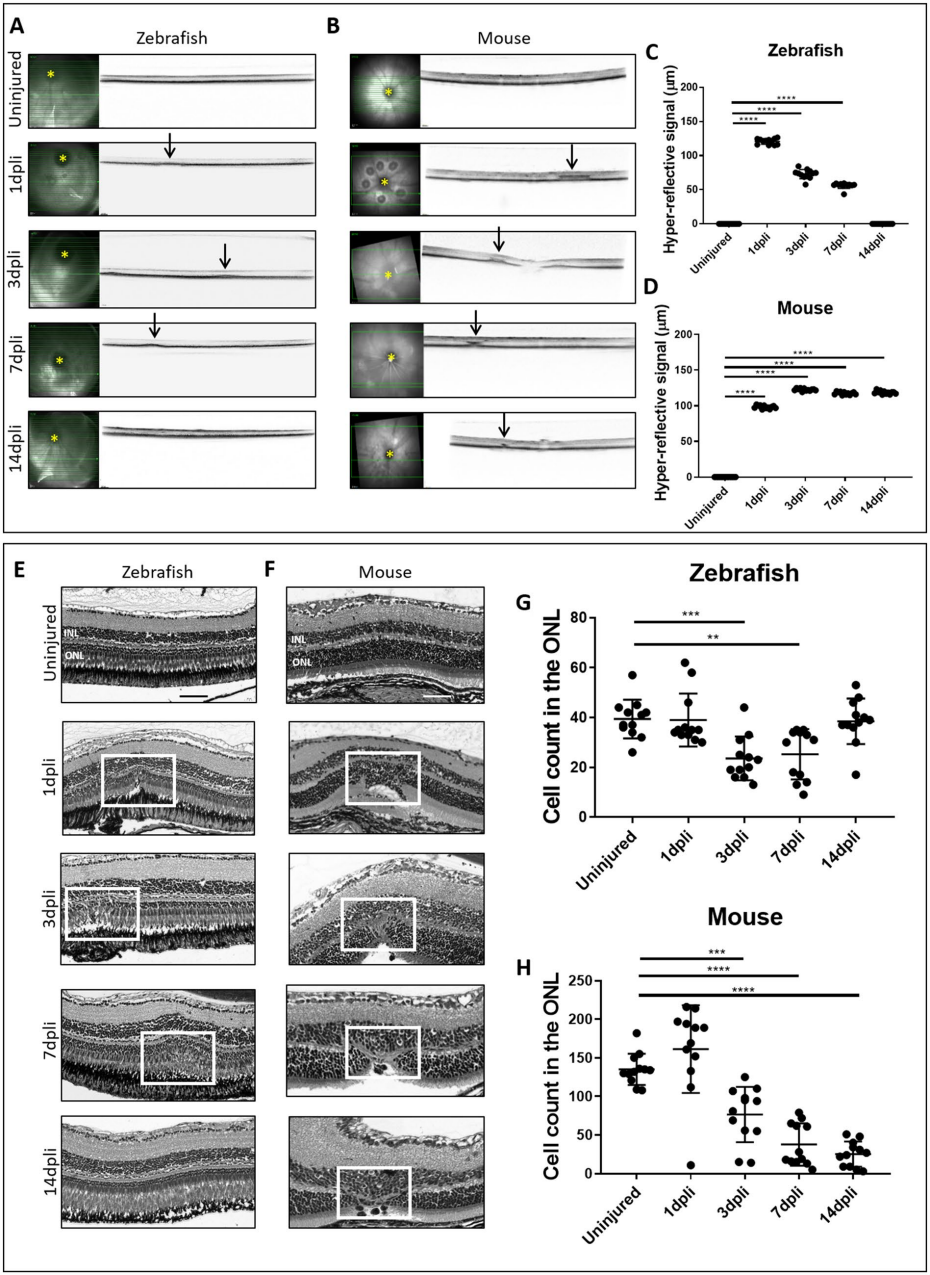
Kinetics of retinal degeneration and regeneration in vivo **a**–**d** OCT and **e**–**h** H&E of the laser area at 1, 3, 7 and 14 dpli. **a**, **b** IR (left) and OCT (right) images of the laser burns from a single animal at different time points. Arrows point to the central lesion depict the injury sites detected as hyper-reflective signal in both animal models. **c**, **d** Quantification of the laser damage width (mean ± SD). Significant differences (****p < 0.001) between controls and the different time points were determined by using a post hoc Bonferroni one-way ANOVA test (*n* = 12). Representative scans were selected (bold green line). **e**, **f** Shown are zebrafish and mouse H&E-stained sections of uninjured and injured retinas at different time points. The damaged area corresponds to photoreceptor layer discontinuity and cavity formation in the ONL and in the subretinal space (white frame). **g**–**h** Quantification of cell nuclei in the ONL in uninjured and injured retinas. The analyzed length of the retina was 50 or 100 µm, corresponding to the induced laser burn size, respectively, in zebrafish and in mouse. Significant differences in structural changes (**p < 0.01, ***p < 0.001 and ****p < 0.0001) between controls and the different time points were determined by using a post hoc Bonferroni one-way ANOVA test in both groups (*n* = 12). *INL* inner nuclear layer; *ONL* outer nuclear layer; *optic nerve head. Scale bar of H&E pictures equals 50 μm

To study the regenerative ability of zebrafish and murine retina, we induced focal damage. The site of damage was visualized by OCT at 1, 3, 7, and 14 dpli. All time points were compared with the uninjured contralateral eye (Uninjured; Fig. 1a–d). At 1 dpli a diffuse hyper-reflective signal was detected in the outer retina (Fig. 1a, b). It extended from the RPE (retinal pigment epithelium) to the OPL (outer plexiform layer). At 3 dpli, the hyper-reflective signal became more dense and localized in the ONL in both animal models. However, in zebrafish, the dimension of the hyper-reflective signal started diminishing from 3 dpli, while in mouse it started increasing from the same time point (Fig. 1a, b). In zebrafish, following the first week (7 dpli), we observed only a small hyper-reflective signal. By 14 dpli, the laser spots were no longer detectable in the IR reflectance mode and OCT (Fig. 1a, c). Conversely, in mouse, a hyper-reflective signal was still visible in the outer retina on 14dpli (Fig. 1b, d).

To confirm the OCT data, we performed H&E staining at 1, 3, 7 and 14 dpli (Fig. 1e–h). Similar to the OCT results (Fig. 1a–d), no difference was detected between murine and zebrafish retina during the first three days. Morphologic changes were consistently observed at 1 dpli with disorganization of the photoreceptor layer and with a cavity formation in the ONL and in the subretinal space (Fig. 1e, f). Indeed, there was a loss of nuclei within the ONL in the damaged area between 1 and 3 dpli (white frame; Fig. 1e, f). In zebrafish, the maximum photoreceptor loss was found at 3 dpli and the retina returned to its normal pattern in the damaged area by 14 dpli (Fig. 1e, g). However, in the murine retina, the average lesion size continued to increase. This caused a massive loss of nuclei within the ONL that persisted until 14 dpli (Fig. 1f, h).

### Upregulation of the Canonical TGFβ Pathway and TGFβ3 Isoform in Müller Cells During Retinal Degeneration/Regeneration in Zebrafish

Both canonical and non-canonical signaling activated by TGFβ isoforms play crucial roles in wound healing and tissue regeneration across vertebrates. The ultimate outcome of this pathway depends on a delicate balance of ligand levels.

**Fig. 2:**
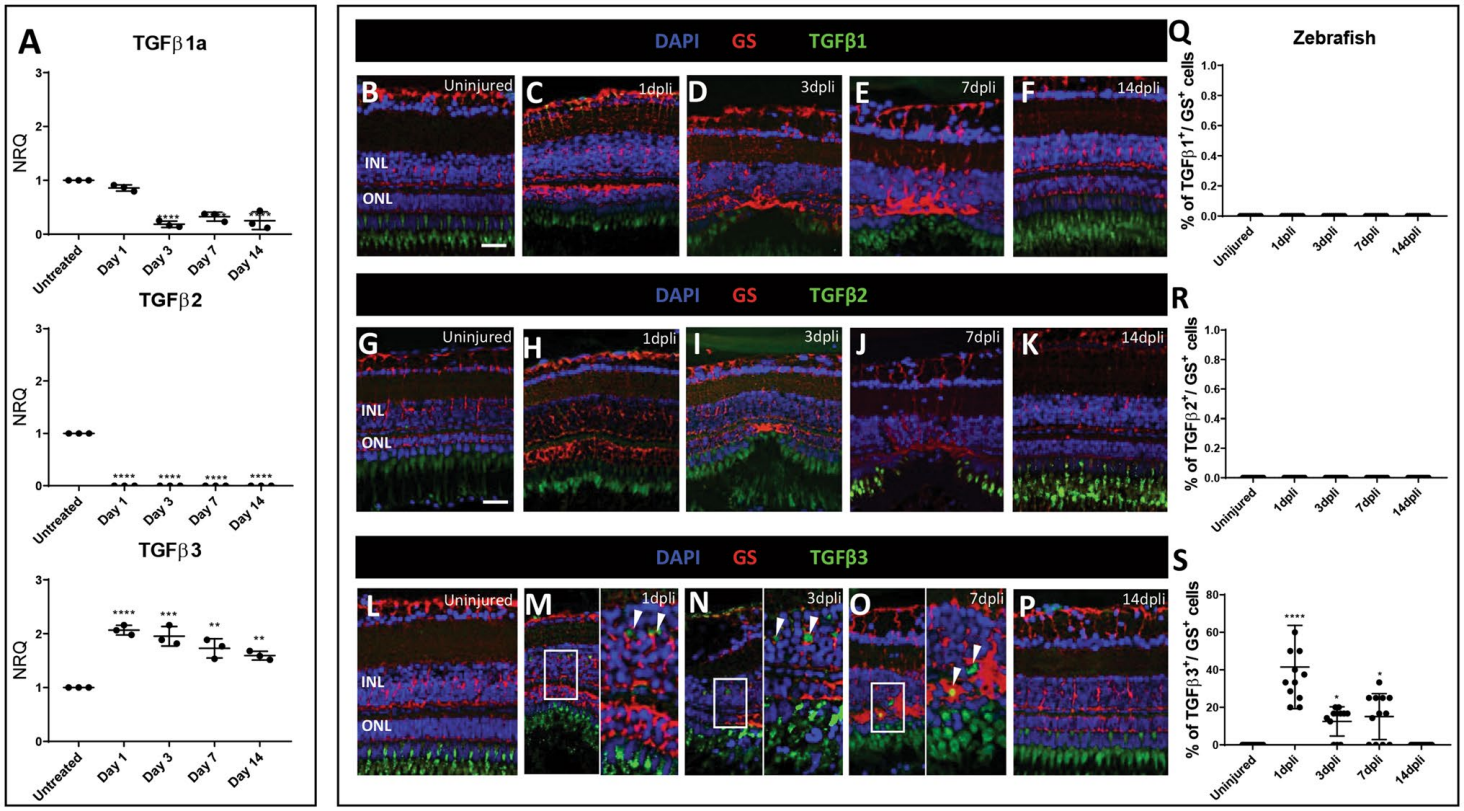
Expression TGFβ isoforms in zebrafish Müller cells: **a** qRT-PCR analyses for *tgfβ1*, *tgfβ*2 and *tgfβ*3 isoforms expressed throughout the lysate of zebrafish whole retinas. For each *tgfβ* isoform, the untreated retinas were always chosen as calibrator [NRQ (*normalized relative quantification)* = 1]. Data are presented as mean + SEM. Graphs show NRQ. **b**–**f** Detection of Tgfβ1 isoform in Müller cells after laser induction and in uninjured zebrafish. Shown are retinal sections stained for GS (red) and Tgfβ1 (green). Cell nuclei were counterstained with DAPI (blue). **g**–**k** Immunofluorescence for GS (red) and Tgfβ2 (green) of a retinal section. Cell nuclei were counterstained with DAPI (blue). **l**–**p** Detection of Tgfβ3 isoform in Müller cells after laser induction and in uninjured zebrafish. Shown are retinal sections stained for GS (red) and Tgfβ3 (green). Cell nuclei were counterstained with DAPI (blue). **m**–**o** Zoomed-in view of GS^+^/Tgfβ3^+^ cells (right side) of the area defined by a white frame (left side). White arrowheads mark double-positive cells. **q**–**s** Histogram illustrating the mean ± SD of the number of Tgfβ1^+^, Tgfβ2^+^ and Tgfβ3^+^ cells normalized by the total number of GS^+^ cells expressed in percentage. Significant differences (*p < 0. 1, ****p < 0.0001) between uninjured and injured zebrafish were determined using a post hoc Bonferroni one-way ANOVA test (*n* = 12). *INL* inner nuclear layer; *ONL* outer nuclear layer. Scale bar of the images equals 50 μm, while in the inserts corresponding to 150 μm

Thus, we investigated *tgfβ1a*, *tgfβ2*, and *tgfβ3* gene expressions in the lysate of zebrafish whole retinas following photoreceptor damage at 1, 3, 7 and 14 dpli by qRT-PCR (Fig. 2a). Regarding *tgfβ1a* and *tgfβ2*, we noticed an opposite regulation compared with *tgfβ3*. *Tgfβ1a* expression was downregulated starting from 3 dpli, whereas *tgfβ2* was already downregulated from 1 dpli. Both gene expressions remained at a low level until 14 dpli in the zebrafish retina. Contrarily, *tgfβ3* was already upregulated starting from 1 dpli and remained upregulated at all time points analyzed (Fig. 2a).

Since fish injury-responsive Müller cells are able to adopt stem cell properties to restore the retina (Wan and Goldman 2016), we determined changes of the three TGFβ isoforms in GS^+^ Müller cells by immunofluorescence analysis at 1, 3, 7 and 14 dpli (Figs. 2b–d; S1a–e). Neither Tgfβ1 nor Tgfβ2 signal was detected in Müller cells after injury throughout the experiment (Fig. 2b–k, q, r). Tgfβ3 was upregulated in Müller cells from 1 dpli within the damaged area (Fig. 2m–p, s). The maximum Tgfβ3 expression was seen at 1 dpli confirming the qRT-PCR data (Fig. 2m, s). At 14 dpli, the regeneration was completed and Tgfβ3 signal was comparable to the controls (Fig. 2l, p, s).

**Fig. 3:**
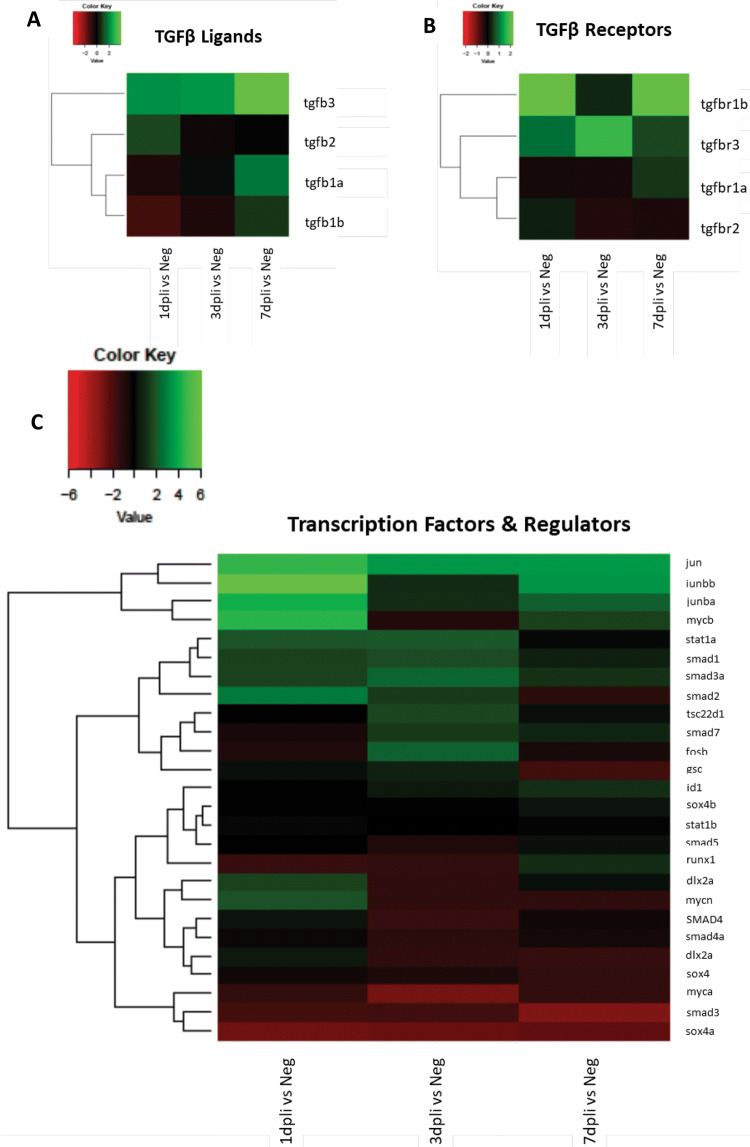
Pathway analysis of cycling gfap:gfap-GFP zebrafish cells showing the activation of canonical signaling. **a**–**c** Heatmaps of differentially expressed Tgfβ receptor, ligand, transcription factor and regulator genes in sorted cycling Müller cells. Data are expressed as fold-changes compared to negative controls (cycling Müller from uninjured zebrafish retinas)

The outcome of TGFβ pathway is highly context-dependent. It results in a complex network of contributing factors, including the levels of signaling mediators within the cell, the extent of activation of canonical versus non-canonical signaling, and the extent to which both of these branches of TGFβ pathway crosstalk with signaling inputs via other receptor systems, both in the cytoplasm and in the nucleus. Herein, we studied the intracellular cascade that evokes the activation of canonical and non-canonical signaling in cycling (S and G2/M phases) Müller cells during regeneration in zebrafish. We compared the gene expression profile of cycling gfap:gfap-GFP cells sorted at 1, 3 and 7 dpli with cycling gfap:gfap-GFP cells from uninjured controls using transcriptome analysis (Fig. 3a–c). Pathway analysis revealed an association of cycling gfap:gfap-GFP cells with activation of canonical signaling via TGFβ3 in zebrafish. Thereby, the maximum gene expression of *tgfb3* was detected at 7 dpli when Müller cells were restoring the damaged area (Fig. 3a). After extracellular activation, Tgfβ3 ligand binds to the membranous tgfbr3 (TGFβ3 receptor). Indeed, *Tgfbr3* was upregulated mostly at 3 dpli (Fig. 3b). Simultaneously with TGFβ3 activation, we also observed an upregulation of activin receptors (*Acvr2aa, Acvr2ab, and Acvrl1*; Fig. S2A) and ligands (*Inhbab, Inhbaa, and Inha*; Fig. S2B) throughout the experiment. Furthermore, TGFβ-dependent signaling can activate or repress hundreds of target genes through the interaction of SMADs (small mothers against decapentaplegic) with various transcription factors (Neuzillet et al. 2014). We analyzed the mRNA levels of transcription factors and regulators of TGFβ pathway (Fig. 3c). *Smad1*, *smad2*, *smad3a* were mainly detected at 3 dpli, while Jun proto-oncogene family genes (*jun*, *junbb*, *junba*) and *mycb* were upregulated at 1 and 7 dpli (Fig. 3c). Finally, inhibitor and cofactors of TGFβ pathway were investigated. We found upregulation of *thbs1* (thrombospondin 1) and *tgfβi* (TGFβ-induced protein), a TGFβ-activating protein (Seliger et al. 2013; Yun et al. 2002), at 1 and 3 dpli. *Bambia* (BMP and activin membrane bound inhibitor a), involved in wound healing by increasing the cell-extracellular matrix interactions (Aitkenhead et al. 2002), was also upregulated in zebrafish Müller cells at 7 dpli (Fig. S2c).

### TGFβ1 and TGFβ2 Expression is Not Linked to TGFβ Canonical Pathway in Murine Müller Cells During Retinal Degeneration/Gliosis

TGFβ pathway is involved in many different biological processes during tissue repair (Grande 1997). We investigated TGFβ isoforms and the activation of canonical or non-canonical TGFβ signaling during glial scar formation.

**Fig. 4:**
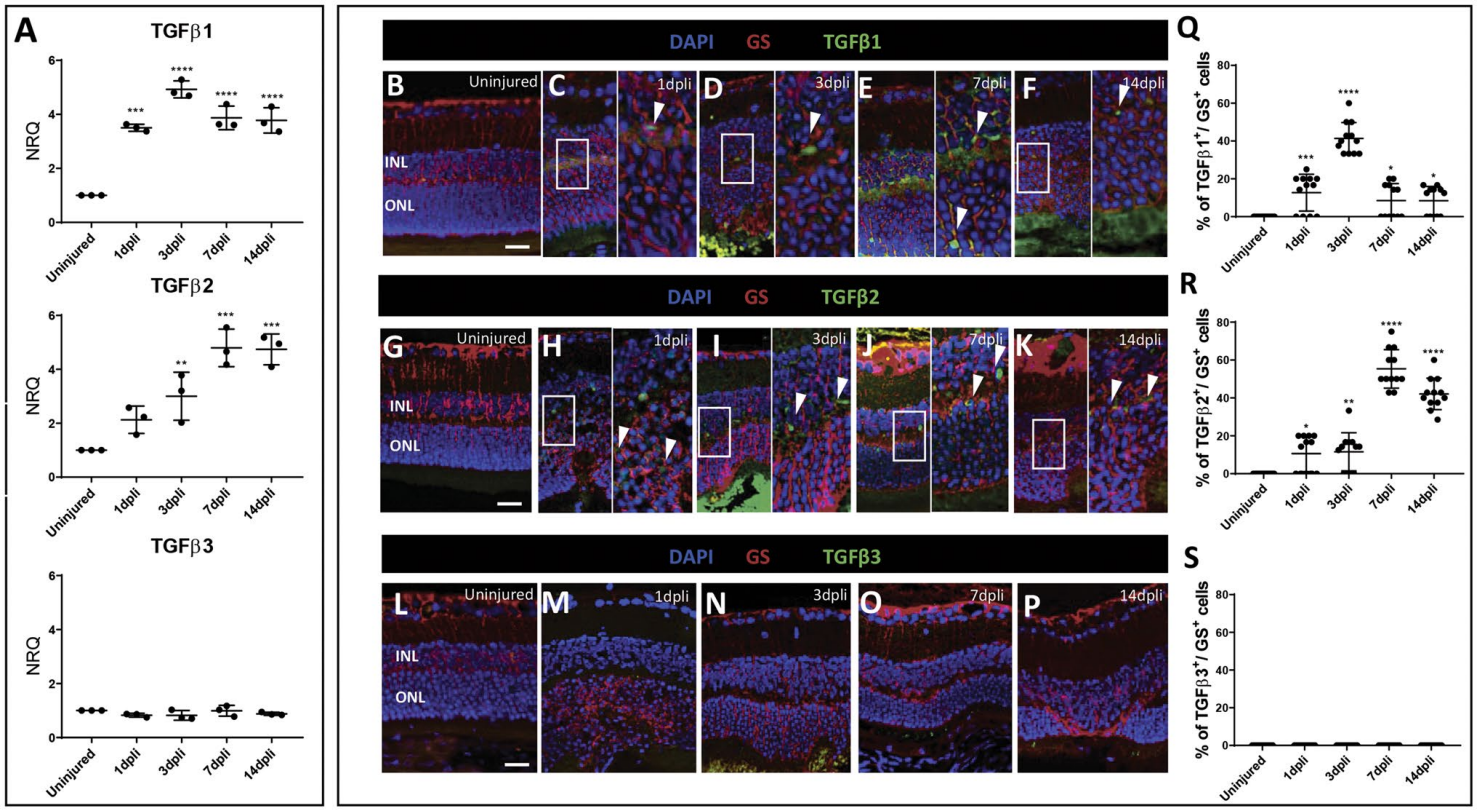
Expression TGFβ isoforms in murine Müller cells. **a** qRT-PCR analyses for *Tgfβ1*, *Tgfβ2* and *Tgfβ3* isoforms expressed in the entire retinas. For each TGFβ isoform, the untreated retinas were always chosen as calibrator [NRQ (*normalized relative quantification)* = 1]. Data are presented as mean + SEM. Graphs show NRQ. **b**–**f** Detection of TGFβ1 isoform in Müller cells after laser induction and in uninjured mice. Shown are retinal sections stained for GS (red) and TGFβ1 (green). Cell nuclei were counterstained with DAPI (blue). **g**–**k** Immunofluorescence for GS (red) and TGFβ2 (green) of a retinal section. Cell nuclei were counterstained with DAPI (blue). **l**–**p** Detection of TGFβ3 isoform in Müller cells after laser induction and in uninjured mice. Shown are retinal sections stained for GS (red) and TGFβ3 (green). Cell nuclei were counterstained with DAPI (blue). **c**–**f** Zoomed-in view of GS^+^/TGFβ1^+^ cells (right side) of the area defined by a white frame (left side). **h**–**k** Zoomed-in view of GS^+^/TGFβ2^+^ cells (right side) of the area defined by a white frame (left side). White arrowheads mark double-positive cells. **q**–**s** Histograms illustrating the mean ± SD of the number of TGFβ1^+^, TGFβ2^+^ and TGFβ3^+^ cells normalized by the total number of GS^+^ cells expressed in percentage. Significant differences (*p < 0.05, **p < 0.01, ***p < 0.001 and ****p < 0.0001) between uninjured and injured murine retinas were determined by using a post hoc Bonferroni one-way ANOVA test (*n* = 12). *INL* inner nuclear layer; *ONL* outer nuclear layer. Scale bar of the images equals 50 μm, while in the inserts corresponding to 150 μm

First, we quantified *Tgfβ1*, *Tgfβ2*, and *Tgfβ3* gene expressions in the lysate of murine whole retinas following photoreceptor damage at 1, 3, 7 and 14 dpli by qRT-PCR (Fig. 4a). We noticed a different regulation of *Tgfβ1* and *Tgfβ2* compared to *Tgfβ3*. *Tgfβ1 and Tgfβ2* expression were upregulated throughout the experiment. The maximum *Tgfβ1* expression was detected at 3 dpli, while *Tgfβ2* expression was highest at 7 dpli. In contrast, *Tgfβ3* was not modulated in mouse (Fig. 4a).

Since Müller cell reactivity ultimately results in glial scar formation in mouse, we determined changes of the three isoforms of TGFβ in GS^+^ Müller cells by immunofluorescence analysis at 1, 3, 7 and 14 dpli (Figs. 4b–s; S3). TGFβ1 and TGFβ2 signal were upregulated in Müller cells starting from 1 dpli and were detectable throughout the experiment (Fig. 4c–f, h–k, q, r). Confirming qRT-PCR data (Fig. 4a), the maximum TGFβ1 signal was detected at 3 dpli (Fig. 4d, q), while TGFβ2 signal was highest at 7 dpli (Fig. 4j, r). No GS^+^ cells expressed TGFβ3 throughout the experiment (Fig. 4l–p, s).

**Fig. 5:**
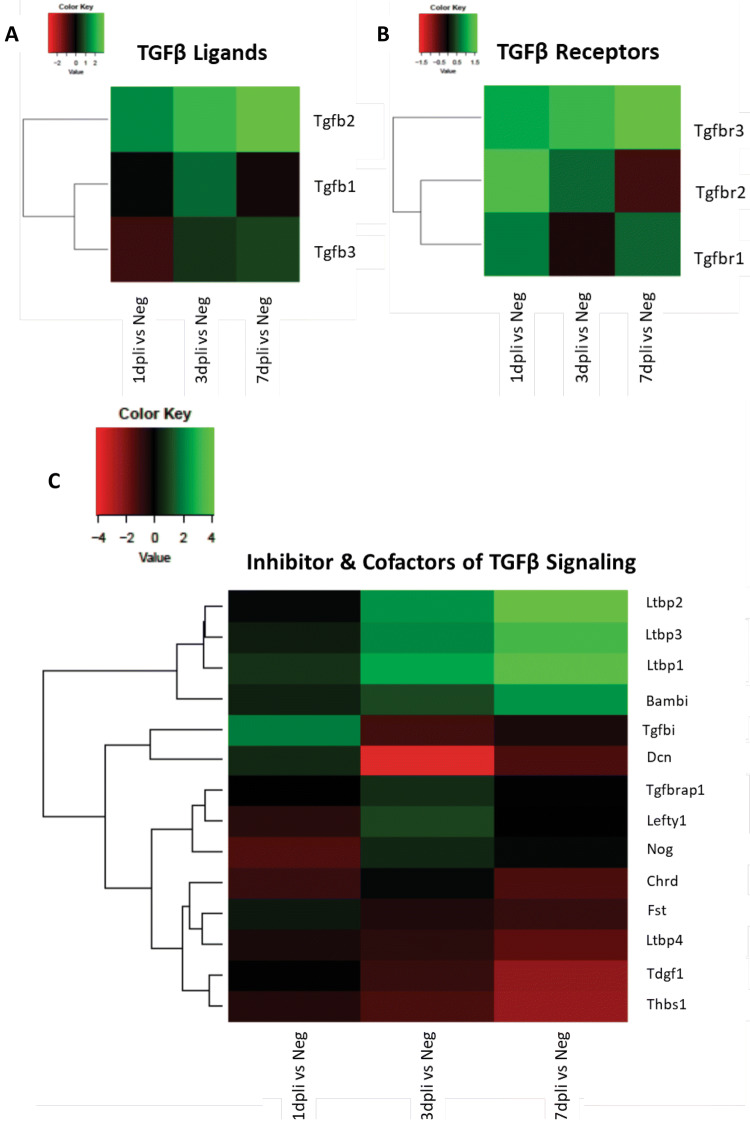
Gene expression profile of cycling Rlbp1:GFP^+^ murine cells. **a**–**c** Heatmaps of TGFβ receptors, ligands, inhibitor, and cofactors of TGFβ signaling differentially expressed genes in sorted cycling Müller cells. Data are expressed as fold-changes compared to negative controls (cycling Müller cells from uninjured murine retinas)

Accordingly, we investigated the intracellular cascade that evokes the activation of canonical and non-canonical signaling in the Müller cells after injury. We compared the gene expression profile of cycling Rlbp1:GFP^+^ cells sorted at 1, 3 and 7 dpli with cycling Rlbp1:GFP^+^ cells from uninjured controls using transcriptome analysis (Fig. 5a–c).

Data revealed a link between cycling Rlbp1:GFP^+^ cells and the activation of TGFβ1 and TGFβ*2*. TGFβ1 ligand was upregulated at 3 dpli (Fig. 5a) and *Tgfbr1* expression was upregulated in cycling Müller cells at 1 and 7 dpli (Fig. 5b). Throughout the experiment, expression of TGFβ*2* continually increased, while its receptor continually decreased (Fig. 5a, b). We also observed an upregulation of *BMP* (bone morphogenic protein) receptors (AMHR2*,* BMPR1a*,* BMPR1b*,* BMPR2; Fig. S4a) and its ligands (Fig. S4b). In particular, we detected an upregulation of BMP2 and BMP7; both are known inhibitors of the TGFβ pathway. Furthermore, we analyzed transcription factors and regulators of TGFβ pathway. Solely, *Tsc22d1* (TGFβ stimulated clone 22d1) was significantly upregulated in mouse and no activation of the Smad cascade was initiated (Fig. S4c). Finally, we investigated inhibitors and cofactors of TGFβ pathway. We found an upregulation of three Ltbp (latent transforming growth factor β binding protein) isoforms (*Ltbp1*, *Ltbp2* and *Ltbp3*) starting at 1 dpli with their maximum expression at 7 dpli (Fig. 5c).

### Activation of Mitogen-Activated Protein Kinase (MAPK) Pathway in Müller Cells After Injury in Both Mouse and Zebrafish

TGFβ can signal in a non-canonical fashion. We investigated the activation of p38MAPK signaling pathway during gliosis in our murine model. All analyses were also performed in zebrafish and the outcomes from both animal models were compared accordingly.

**Fig. 6:**
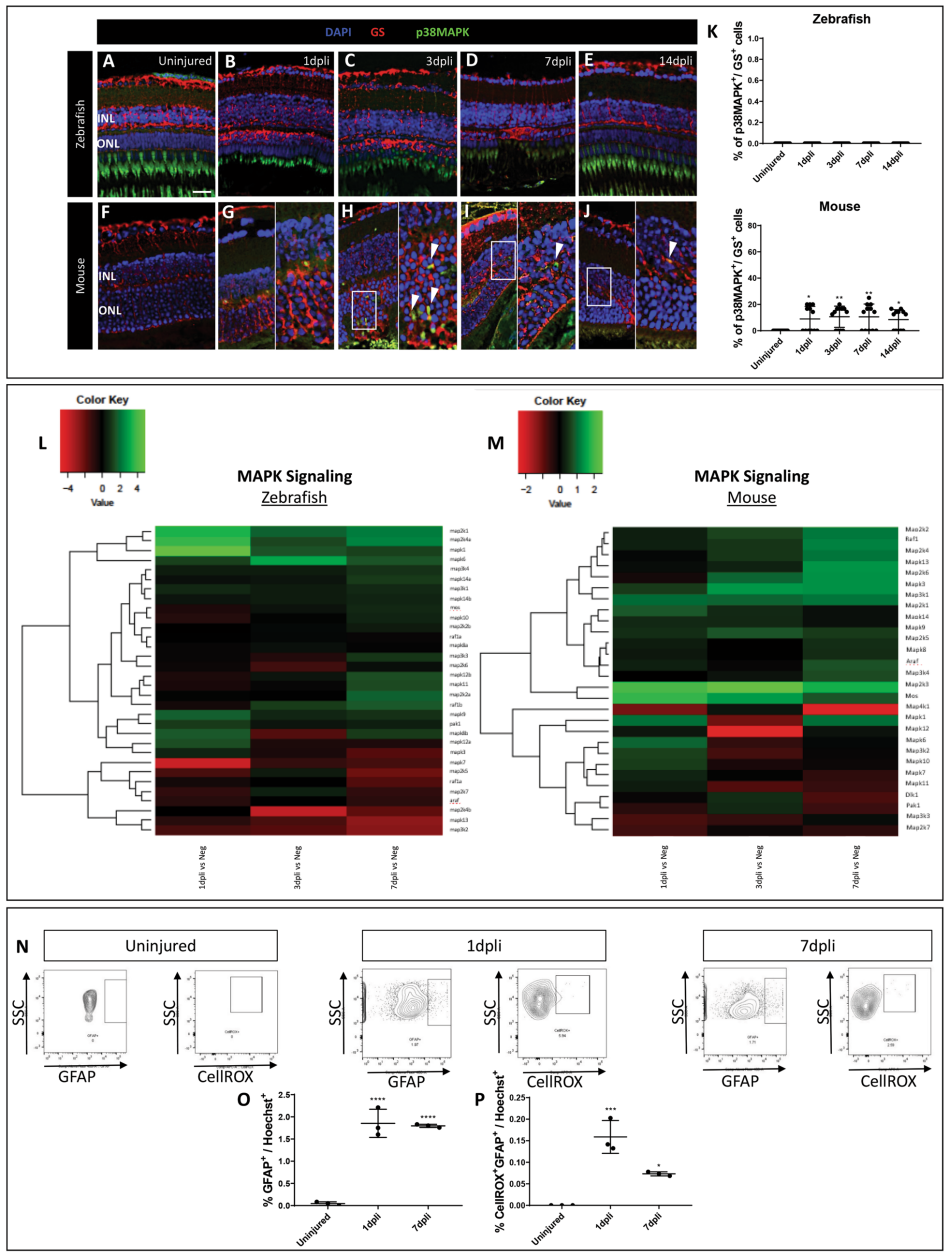
Müller cells contribute to glial scar formation via MAPK signaling pathway. **a**–**j** Analysis of p38 MAPK^+^ Müller cells in zebrafish and murine retinas at 1, 3, 7 and 14 dpli. Shown are retinal sections stained for GS (red) and p38 MAPK (green). Cell nuclei were counterstained with DAPI (blue). **h**–**j** Zoomed-in view of GS^+^/p38 MAPK^+^ cells (right side) of the area defined by a white frame (left side). White arrowheads mark double-positive cells. **k** Histograms illustrating the mean ± SD of the number of p38 MAPK^+^ cells normalized by the total number of GS^+^ cells expressed in percentage in both animal models. Significant differences (*p < 0.05; **p < 0.01) between uninjured and injured animals were determined by using a post hoc Bonferroni one-way ANOVA test (*n* = 12). *INL* inner nuclear layer; *ONL* outer nuclear layer. Scale bar of the images equals 50 μm, while in the inserts corresponding to 150 μm. **l**–**m** Heatmaps of MAPK pathway differentially expressed genes in sorted cycling Müller cells. Data are expressed as fold-changes compared to negative controls (cycling Müller cells from uninjured retinas). **n** Flow cytometry analysis of ROS production in GFAP^+^ Müller cells in mouse. Müller cells were gated as GFAP^+^ cells (left) and were further gated as CellROX Deep Red^+^ (right). **o**–**p**) Histograms illustrating the mean ± SD of the number of GFAP^+^ Müller cells and GFAP^+^/CellROX Deep Red^+^ cells normalized by the total number of Hoechst^+^ cells expressed in percentage at 1 and 7 dpli. Significant differences (*p < 0.05, ***p < 0.001 and ****p < 0.0001) between uninjured, 1 and 7 dpli were determined by using a post hoc Bonferroni one-way ANOVA test (*n* = 12). *SSC* side scatter

Immunofluorescence analysis was performed to determine changes of p38MAPK in GS^+^ Müller cells 1, 3, 7 and 14 dpli in both animal models (Figs. 6a–k; S5a–c). p38MAPK signal was not detectable in zebrafish Müller cells throughout the experiment (Fig. 6a–e, k). Contrariwise, p38MAPK signal was upregulated starting from 1 until 14 dpli in murine Müller cells (Fig. 6g–j, k). Maximum p38MAPK expression was evident at 3 and 7 dpli within the damaged area (Fig. 6h, i, k).

Accordingly, we compared the gene expression profile of zebrafish cycling gfap:gfap-GFP cells sorted at 1, 3 and 7 dpli with cycling gfap:gfap-GFP cells from uninjured retinas using transcriptome analysis (Fig. 6l). In mouse, we compared the gene expression profile of cycling Rlbp1:GFP^+^ cells sorted at 1, 3 and 7 dpli with negative controls (cycling Müller cells from uninjured retinas) using transcriptome analysis (Fig. 6m). Pathway analysis revealed an association of murine cycling Rlb1:GFP^+^ cells with activation of MAPK pathway. Particularly, *Map2k6* and *Map3k4*, which can phosphorylate p38 isoforms (*p38α, p38β, p38γ* and *p38δ*), were upregulated at 7 dpli (Fig. 6m). Furthermore, *Map2k3*, which is activated by mitogenic and environmental stress (Raingeaud et al. 1996), was upregulated throughout the experiment in mouse with maximum expression detected at 3 dpli (twofold; Fig. 6m). *Mos (*proto-oncogene serine/threonine-protein kinase Mos), upstream activator of MAPK (Choi et al. 1996), was upregulated starting from 1 until 3 dpli with the maximum gene expression at 1 dpli (1.5-fold; Fig. 6m).

Finally, TGFβ1 signaling through Smad pathway is known to be responsible for the induction of many TGFβ responsive genes. Emerging evidence indicates that ROS mediate TGFβ signaling through different pathways including MAPK pathway (Rhyu et al. 2005). Therefore, we analyzed ROS production in activated Müller cells (GFAP^+^) and compared with the uninjured contralateral eye (Fig. 6n–p). ROS production was especially detected in activated murine Müller cells at 1 dpli (Fig. 6n, p), in parallel with the activation of MAPK signaling pathway after injury.

### Activation of MAPK Signaling Pathway During Fibrotic-Like Response in Both Murine and Zebrafish Müller Cells

TGFβ1 is a potent inducer of plasminogen activator inhibitor 1 (PAI1) expression leading to inhibition of protease-dependent proteolytic activity and accumulation of extracellular matrix, resulting in fibrosis (Omori et al. 2016). Furthermore, ROS upregulates expression of PAI1 (Gorlach et al. 2003). Thus, we investigated the activation of PAI1 during gliosis. All analyses were performed also in zebrafish and the outcomes from both animal models were compared accordingly.

**Fig. 7:**
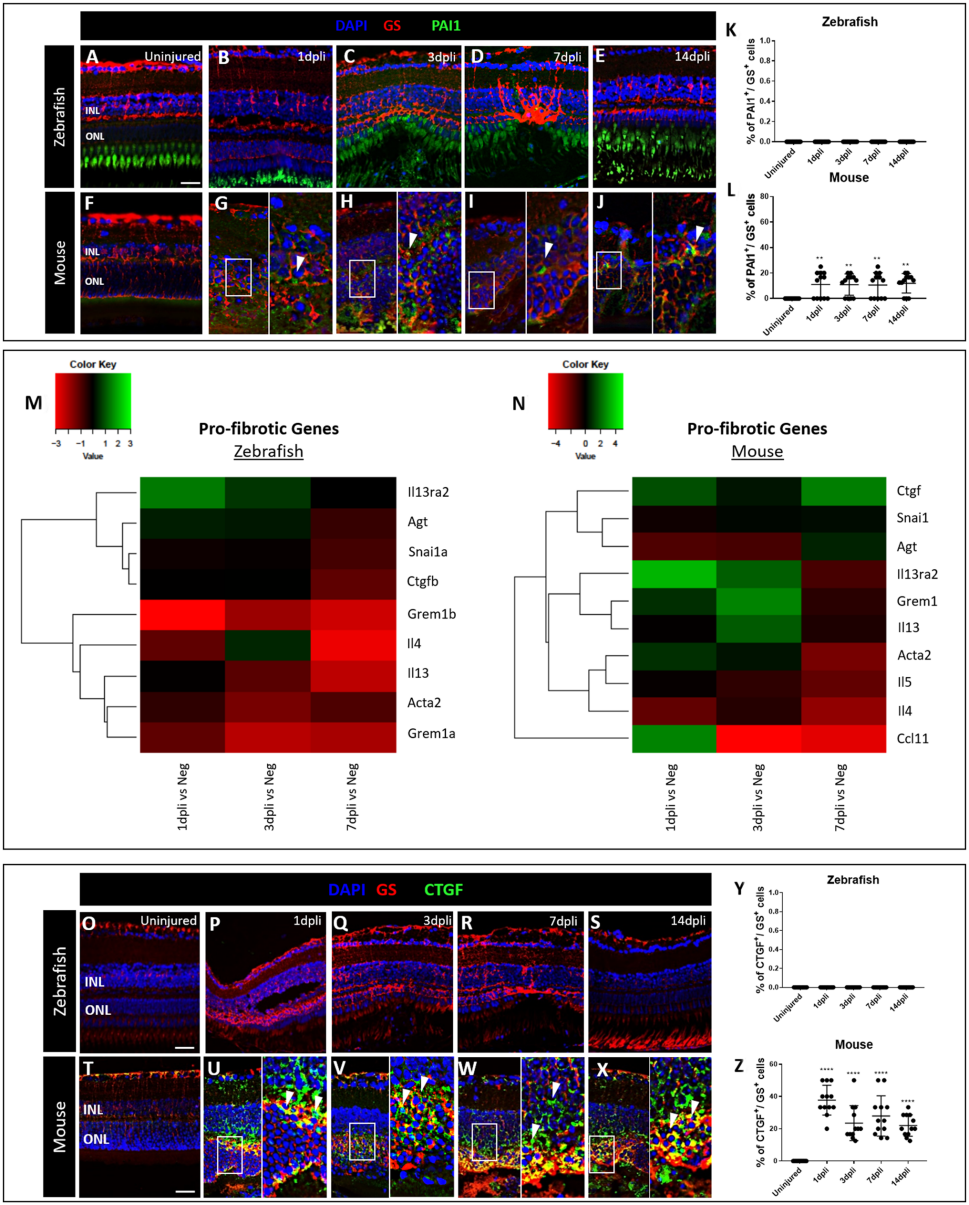
PAI1 upregulation in mouse is associated with retinal gliosis. **a**–**j** Analysis of PAI1^+^ Müller cells in zebrafish and murine retinas at1, 3, 7 and 14 dpli. Shown are retinal sections stained for GS (red) and PAI1 (green). Cell nuclei were counterstained with DAPI (blue). **g**–**j** Zoomed-in view of GS^+^/PAI1^+^ cells (right side) of the area defined by a white frame (left side). White arrowheads mark double-positive cells. **k**–**l** Histograms illustrating the mean ± SD of the number of PAI1^+^ cells normalized by the total number of GS^+^ cells expressed in percentage. Significant differences (**p < 0.01) between uninjured and injured animals were determined using a post hoc Bonferroni one-way ANOVA test (*n* = 12). **m**–**n** Heatmaps of pro-fibrotic genes differentially expressed genes in sorted cycling Müller cells. Data are expressed as fold-changes compared to negative controls (cycling Müller cells from uninjured retinas). **o**–**x** Detection of CTGF in Müller cells after laser induction and in uninjured retinas in both animal models. Shown are retinal sections stained for GS (red) and CTGF (green). **u**–**x** Zoomed-in view of GS^+^/CTGF^+^ cells (right side) of the area defined by a white frame (left side). White arrowheads mark double-positive cells. **y**–**z** Histograms illustrating the mean ± SD of the number of CTGF^+^ cells normalized by the total number of GS^+^ cells expressed in percentage. Significant differences (****p < 0.0001) between uninjured and injured animals were determined by using a post hoc Bonferroni one-way ANOVA test (*n* = 12). *INL* inner nuclear layer, *ONL* outer nuclear layer. Scale bar of the images equals 50 μm, while in the inserts corresponding to 150 μm

Immunofluorescence analysis was performed to determine changes of PAI1 in GS^+^ Müller cells at 1, 3, 7 and 14 dpli in both animal models (Figs. 7a–l; S6a–c). PAI1 signal was not visible in zebrafish Müller cells throughout the experiments (Fig. 7a–e, l). Contrariwise, PAI1 signal was upregulated in murine Müller cells from 1 dpli throughout 14 dpli (Fig. 7g–l), suggesting the suppression of fibrinolysis via PAI1 production.

Accordingly, we investigated pro- and anti-fibrotic genes to clarify if the induced gliosis is a fibrotic-like process. In mouse, we compared the transcriptome of cycling Rlbp1:GFP^+^ cells sorted at 1, 3, and 7 dpli to uninjured controls. In zebrafish, we compared the gene expression profile of cycling gfap:gfap-GFP cells sorted at 1, 3 and 7 dpli with controls using transcriptome analysis (Figs. 7m, n, S7a, b). Data revealed a downregulation of most pro-fibrotic genes in the cycling gfap:gfap-GFP cells in zebrafish (Fig. 7m). However, murine cycling Rlb1:GFP^+^ cells showed an upregulation of *Ctgf* (connective tissue growth factor), *Il13ra2* (interleukin-13 receptor subunit alpha-2), *Grem1* (gremlin 1), *Il13* (interleukin-13) and *Ccl11* (eosinophil chemotactic protein) genes associated with development and progression of fibrosis (Fig. 7n). On the other hand, we detected an upregulation of the anti-fibrotic gene, *il10* (interleukin 10), exclusively in zebrafish (Fig. S7a).

To confirm the mRNA analyses, we performed immunofluorescence staining for CTGF in GS^+^ Müller cells at 1, 3, 7 and 14 dpli in both animal models (Figs. 7o–s; S8a–c). CTGF signal was not visible in zebrafish Müller cells throughout the experiments (Fig. 7o–s, y). Contrariwise, the signal was upregulated starting from 1 dpli until the last time point investigated (14 dpli) in mouse only (Fig. 7t–x, z).

### Induction of a Fibrotic-Like Response After Laser Induction in Zebrafish by Anti-fibrinolytic Treatment

To study the role of PAI1 during tissue repair, zebrafish were immersed in TXA water 12 h before damage induction and kept in TXA water until 14 dpli. TXA is a synthetic anti-fibrinolytic substance that blocks the lysine binding sites of plasminogen and thereby leading to PAI1 inhibition (Renckens et al. 2004).

**Fig. 8:**
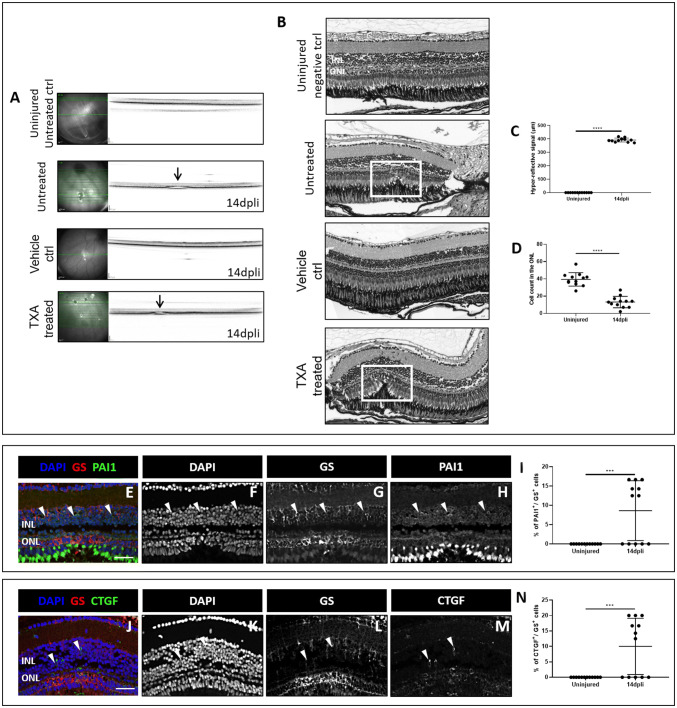
Induction of a fibrotic-like process after injury in TXA-treated zebrafish. **a**, **b** In vivo imaging and **c**, **d** morphological analysis of the laser area in uninjured untreated control (ctrl) and in untreated, vehicle ctrl and TXA-treated zebrafish at 14 dpli. **a** IR (left) and OCT (right) images of the laser burns. Arrowheads point to the central lesion on OCT depict the injury sites detected as hyper-reflective signal in both animal models. **b** Shown are zebrafish H&E-stained retinal sections of uninjured untreated ctrl and of untreated, vehicle ctrl and TXA-treated zebrafish at 14 dpli. The damaged area corresponds to photoreceptor layer discontinuity and cavity formation in the ONL and in the subretinal space (white frame). **c** Quantification of the laser damage width (mean ± SD). Significant differences (***p < 0.001) between uninjured untreated ctrl and untreated, vehicle ctrl and TXA-treated zebrafish at 14 dpli were determined by using two-tailed *t* test (*n* = 12). Representative scans were selected as indicated by the bold green line. **d** Quantification of cell nuclei in the ONL in uninjured and injured retinas at 14 dpli. Significant differences in structural changes after laser damage (***p < 0.001) between uninjured untreated ctrl and untreated, vehicle ctrl and TXA-treated zebrafish at 14 dpli were determined by using two-tailed *t* test (*n* = 12). **e**–**i** Analysis of Müller cell PAI1 expression in the TXA-treated zebrafish retinas at 14 dpli. **e**–**h** Shown are retinal sections at 14 dpli after laser damage induction stained for GS (red) and PAI1 (green). Cell nuclei were counterstained with DAPI (blue). **i** Histogram illustrating the mean ± SD of the number of PAI1^+^ cells normalized by the total number of GS^+^ cells expressed in percentage in the TXA-treated zebrafish. Significant differences (****p < 0.0001) between uninjured and injured animals were determined by using two-tailed *t* test (*n* = 12). **j**–**n** Analysis of Müller cells CTGF expression in the TXA-treated zebrafish retinas at 14 dpli. **j**–**m** Shown are retinal sections at 14 dpli after laser damage induction stained for GS (red) and CTGF (green). Cell nuclei were counterstained with DAPI (blue). **n** Histogram illustrating the mean ± SD of the number of CTGF^+^ cells normalized by the total number of GS^+^ cells expressed in percentage in the TXA-treated zebrafish. Significant differences (***p < 0.001) between uninjured and injured animals were determined by using two-tailed *t* test (*n* = 12). *INL* inner nuclear layer, *ONL* outer nuclear layer. Scale bar of H&E pictures equals 50 μm

The kinetics of retinal regeneration at 14 dpli was evaluated by OCT. A hyper-reflective signal was detected in the ONL in the TXA-treated group (Fig. 8a). To confirm the OCT data, we performed H&E staining at 14 dpli after damage induction (Fig. 8b). According to the model, the outer retina would have re-established its normal banding pattern in the damaged area in zebrafish at that time point. However, in TXA-treated zebrafish, the injury persisted until 14 dpli (Fig. 8b, white frame).

Immunofluorescence analysis was performed to determine changes of PAI1 and CTGF in GS^+^ Müller cells at 14 dpli in the TXA-treated zebrafish. PAI1 signal was upregulated in the TXA-treated zebrafish until 14 dpli (Fig. 8e–i), suggesting suppression of fibrinolysis via TXA treatment. CTGF signal was upregulated at 14 dpli within the damaged area (Fig. 8j–n) as we observed in the murine model (Fig. 7t–z).

## Discussion

TGFβ belongs to a group of pleiotropic cytokines that are involved in a variety of biological processes in the central nervous system (CNS), such as repair and regeneration. In particular, TGFβ pathway affects the CNS immune response, phenotypic modulation of neural cells, scar formation, and modulation of neurotrophic factors (Li et al. 2017). Specific TGFβ isoforms and downstream mediators of canonical and non-canonical signaling play different roles in each of these processes. Here, we found that the canonical pathway is related to regeneration in zebrafish, while the non-canonical signaling is related to tissue repair and gliosis in mouse.

In zebrafish Müller cells, only Tgfβ3 ligand was upregulated. Furthermore, expression of all activin receptors and ligands in Müller cells were increased throughout the experiment, promoting in combination Smad signaling (McLean and Di Guglielmo 2010). Earlier studies showed that *jun* genes are highly expressed during regenerative processes (Ishida et al. 2010). Expression of both zebrafish *junb* orthologues (*junba* and *junbb*) were increased in Müller cells after injury. The Müller cell progenitor marker gene *mycb* (Mitra et al. 2019) was also upregulated during damage response in zebrafish. This suggests that the simultaneous upregulation of *junb* genes and *mycb* at 14 dpli regulate the TGFβ cascade, activating the canonical signaling via TGFβ3 in zebrafish during regenerative response.

While TGFβ3 is activated after injury in the zebrafish, we found TGFβ1 and TGFβ2 were activated in Müller cells in our murine model. Some BMP receptors and ligands were also upregulated throughout the experiment, e.g., BMP2 and BMP7*.* Both genes are known to induce changes of markers typically associated with gliosis (e.g., GS, vimentin, S100β, CNTF) in murine Müller cells (Dharmarajan et al. 2014; Ueki and Reh 2012). Although BMPs can signal through both canonical and non-canonical TGFβ pathway, we did not detect significant upregulation of Smad signaling through transcriptome analysis, which suggested the possible activation of the non-canonical signaling during gliosis in Müller cells. Indeed, several non-canonical Smad-independent signaling have been identified for BMPs. Studies in *Xenopus laevis* associated Bmp2 and Bmp7 upregulation with p38 MAPK activation (Herpin and Cunningham 2007). TGFβ1 increases production of ROS by impairing mitochondrial function (Liu and Desai 2015) and mediates the p38 MAPK pathway (Yu et al. 2002). We also detected upregulation of Ltbp isoforms, *Ltbp1*, *Ltbp2* and *Ltbp3*. Their modulation can be directly mediated by ROS production (Jobling et al. 2006) and activate the p38 MAPK pathway (Sideek et al. 2017) linked to gliosis (Kaminska et al. 2009). Thus, we analyzed p38 MAPK signaling and found evidence of activation of the non-canonical p38 MAPK pathway—likely mediated by TGFβ1 and TGFβ2—during gliotic response in mouse. We also detected increasing upregulation of *Tsc22d1* in Müller cells. In agreement with our findings, *Tsc22* has been shown to sequester *Smad7* from binding to activated *Tgfbr1*, thus hindering Smad7/Smurf-induced ubiquitination and degradation of the receptor (Xu 2011). Additionally, Yan et al. demonstrated that *Tsc22* promotes expression of fibrotic genes (e.g., *αSMA*, *PAI1*, *Fn1*, and *Col1*), contributing to myocardial fibrosis (Yan et al. 2011). So, we investigated the implication of PAI1, a key prognostic marker for fibrotic disease (Ghosh and Vaughan 2012), in zebrafish and murine Müller cells during damage response. PAI1 signal remained upregulated for the course of the experiment only in murine Müller cells. Additionally, many pro-fibrotic genes were overexpressed after injury in murine Müller cells, suggesting that gliosis can be considered a fibrotic-like process.

## Conclusion

Altogether, these results indicate that TGFβ isoforms have different effects on tissue repair, which may be context‐dependent (Gilbert et al. 2016; Morikawa et al. 2016; Klass et al. 2009). TGFβ3 seems to be related to retinal regeneration via canonical signaling upon regulation of *junb* and *mycb* orthologues in zebrafish Müller cells, while TGFβ1 and TGFβ2 seem to be linked to p38 MAPK pathway in the mouse.

*Il10* was the only anti-fibrotic gene differentially expressed between species in Müller cells; it was upregulated in zebrafish and downregulated in murine Müller cells. Earlier studies have shown that *Il10* acting on TGFβ pathway can have therapeutic benefits for preventing and reducing scar formation (Shi et al. 2013). However, relatively little is known about the mechanisms underlying *Il10*-mediated anti-fibrotic and scar-improvement actions and future studies are warranted.

Overall, our comprehensive cross-species transcriptome analysis reveals the activation of different signaling and differential expression of gene regulatory networks that will help to explain why some species, such as zebrafish can regenerate, while others, such as mouse, cannot. It also provides a useful resource for further studies on the development of therapeutic strategies for gliosis.

## Electronic supplementary material

Below is the link to the electronic supplementary material.

Supplementary file1 (PDF 1471 kb)
