# Supplementary material for: Diverse Signaling by TGFβ Isoforms in Response to Focal Injury is Associated with Either Retinal Regeneration or Reactive Gliosis
Source: Cell Mol Neurobiol. 2020 Mar 26;41(1):43–62. doi: 10.1007/s10571-020-00830-5 (PMC7811507; doi:10.1007/s10571-020-00830-5)
Supplement: Supplementary file 1 — Supplementary file1 (PDF 1471 kb) [file 10571_2020_830_MOESM1_ESM.pdf]

Supplementary figures:

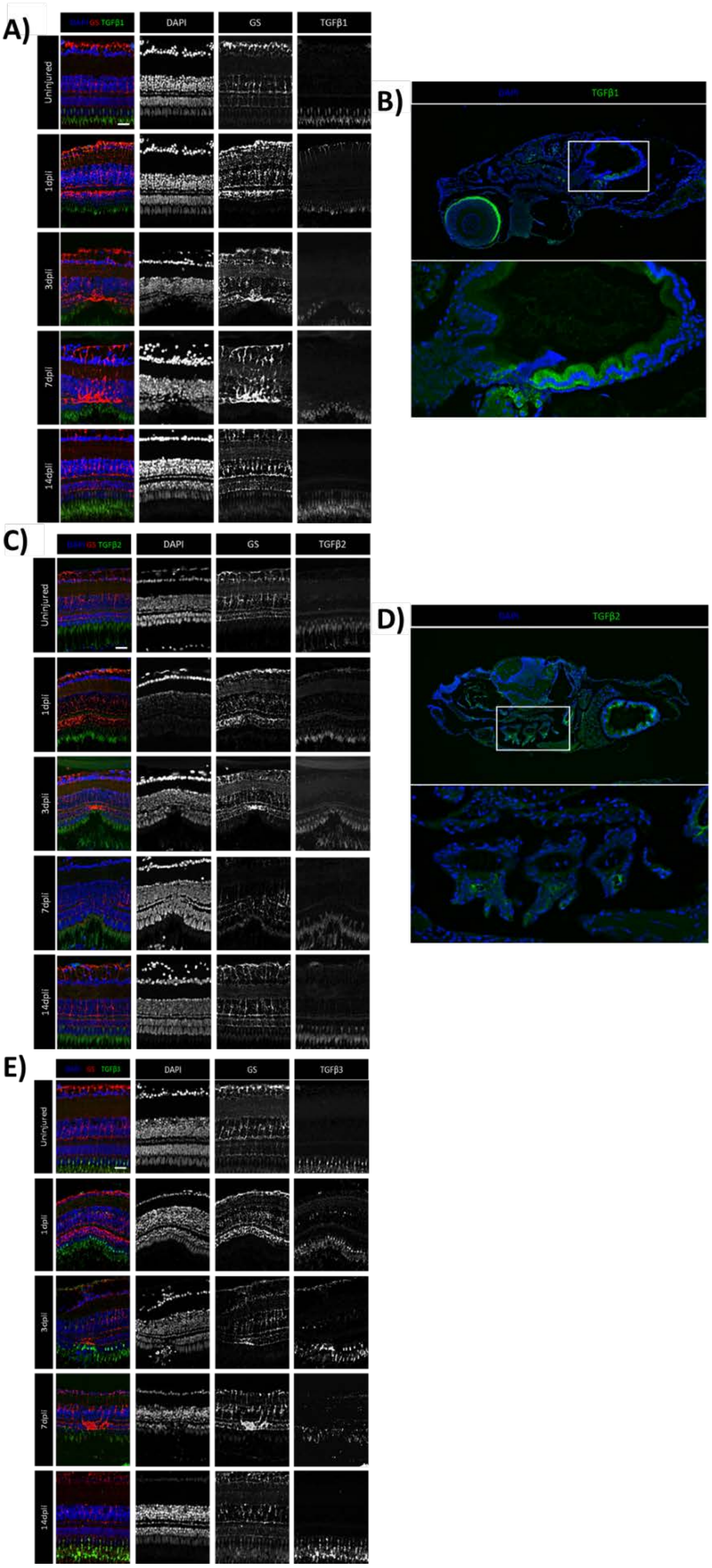

Figure S1: Expression of Tgfβ1, Tgfβ2 and Tgfβ3 in zebrafish Müller cells during laser injury response. (A) Analysis of Müller cell Tgfβ1 expression in Zebrafish retina during laser

injury response and in negative controls (Uninjured). Shown are retinal sections at different time points (1, 3, 7 and 14 dpli) after laser damage induction stained for GS (red) and Tgfβ1 (green). (B) Shown are retinal sections of zebrafish embryo stained for Tgfβ1 (green). (C) Analysis of Müller cell Tgfβ2 expression in the zebrafish retina during laser injury response and in negative controls (Uninjured). Shown are retinal sections at different time points (1, 3, 7 and 14 dpli) after laser damage induction stained for GS (red) and Tgfβ2 (green). (D) Shown are retinal sections of zebrafish embryo stained for Tgfβ2 (green). (E) Analysis of Müller cell Tgfβ3 expression in the zebrafish retina during laser injury response and in negative controls (Uninjured). Shown are retinal sections at different time points (1, 3, 7 and 14 dpli) after laser damage induction stained for GS (red) and Tgfβ3 (green). Cell nuclei were counterstained with DAPI (blue). INL, inner nuclear layer; ONL, outer nuclear layer. Scale bar of the images equals 50 µm.

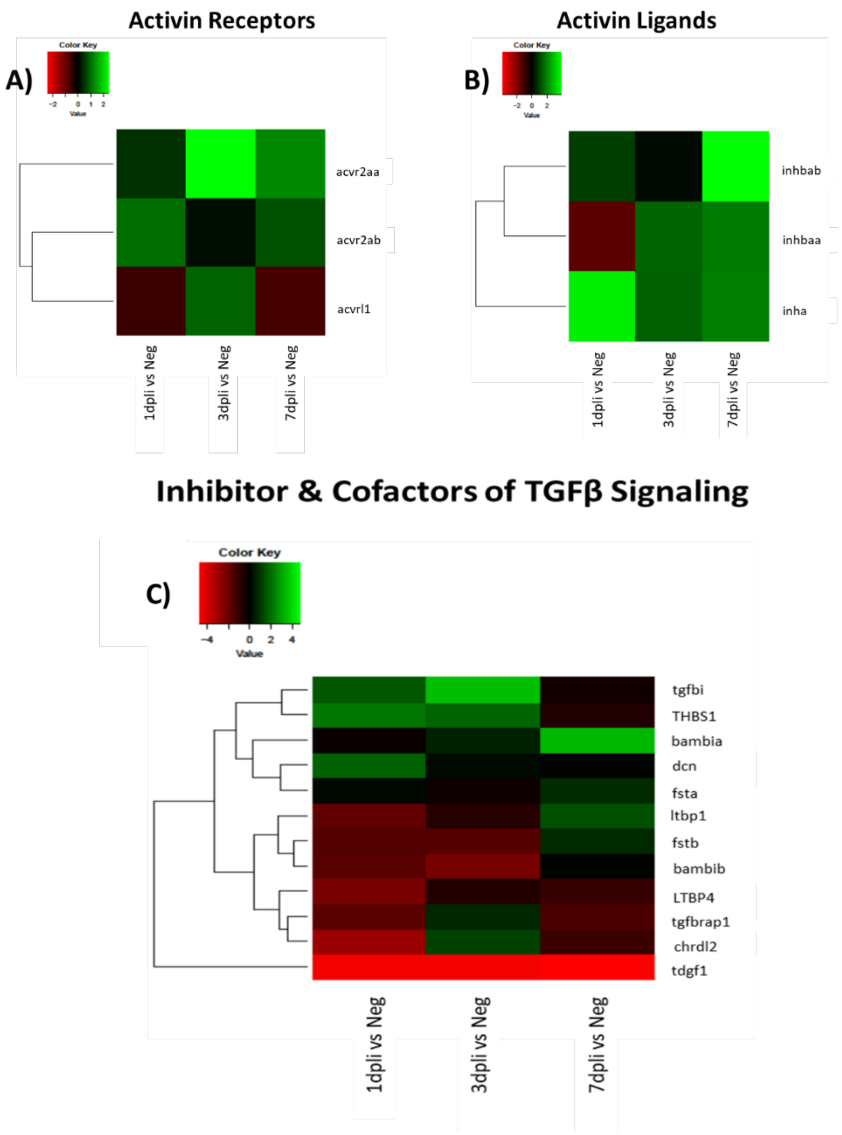

Figure S2: Heatmaps of differentially expressed genes of the TGFβ pathway in sorted proliferating and activated Müller cells in zebrafish. (A) Heatmaps of activin receptors

differentially expressed genes in sorted proliferating and activated Müller cells. (B) Heatmaps of activin ligands differentially expressed genes in sorted proliferating and activated Müller cells. (C) Heatmaps of inhibitor and cofactors of TGFβ signaling differentially expressed genes in sorted proliferating and activated Müller cells. Data are expressed as fold-changes compared to negative controls (uninjured retinas from age-matched, undamaged siblings).

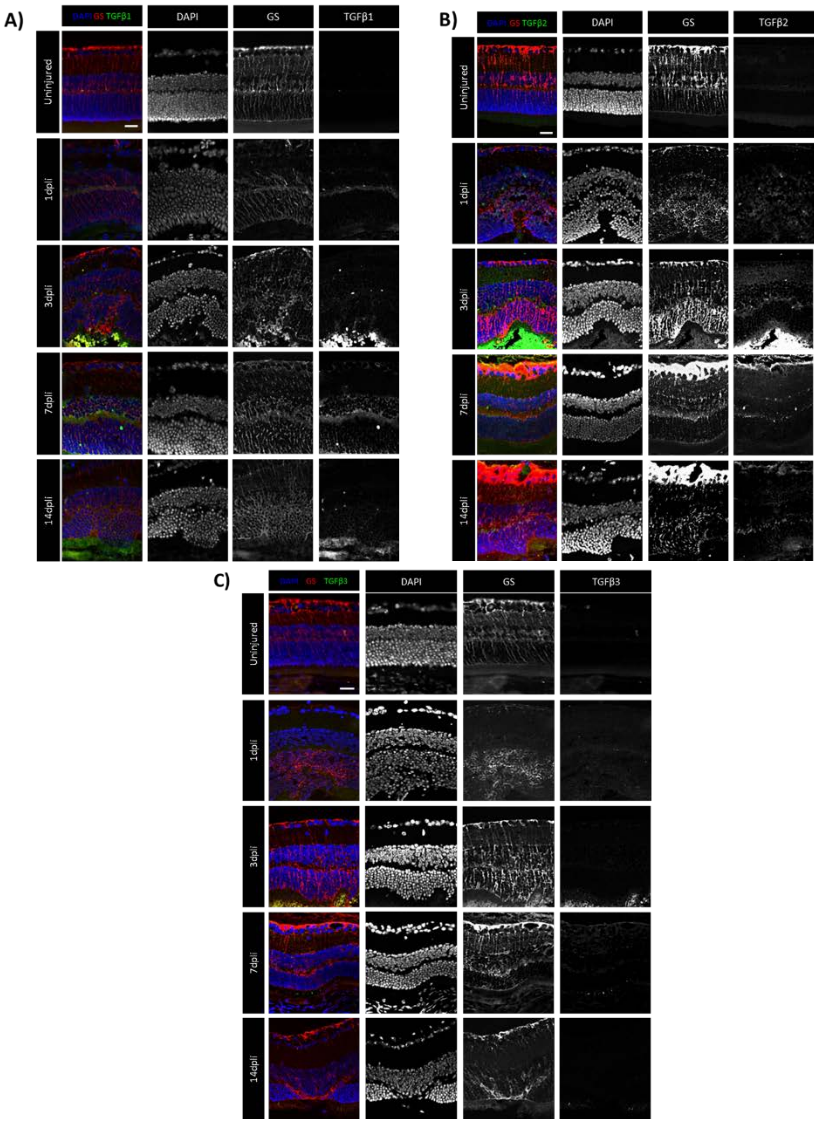

Figure S3: Expression of TGFβ1, TGFβ2 and TGFβ3 in murine Müller cells during laser injury response. (A) Analysis of Müller cells TGFβ1 expression in the murine retina during laser injury response and in negative controls (Uninjured). Shown are retinal sections at different time points (1, 3, 7 and 14dpli) after laser damage induction stained for GS (red) and TGFβ1 (green). Cell nuclei were counterstained with DAPI (blue). INL, inner nuclear layer; ONL, outer nuclear layer. Scale bar of the images equals 50 µm. (B) Analysis of Müller cell TGFβ2 expression in the murine retina during laser injury response and in negative controls (Uninjured). Shown are retinal sections at different time points (1, 3, 7 and 14 dpli) after laser damage induction stained for GS (red) and TGFβ2 (green). Cell nuclei were counterstained

with DAPI (blue). INL, inner nuclear layer; ONL, outer nuclear layer. Scale bar of the images equals 50 µm. (C) Analysis of Müller cell TGFβ3 expression in the murine retina during laser injury response and in negative controls (Uninjured). Shown are retinal sections at different time points (1, 3, 7 and 14 dpli) after laser damage induction stained for GS (red) and TGFβ3 (green). Cell nuclei were counterstained with DAPI (blue). INL, inner nuclear layer; ONL, outer nuclear layer. Scale bar of the images equals 50 µm.

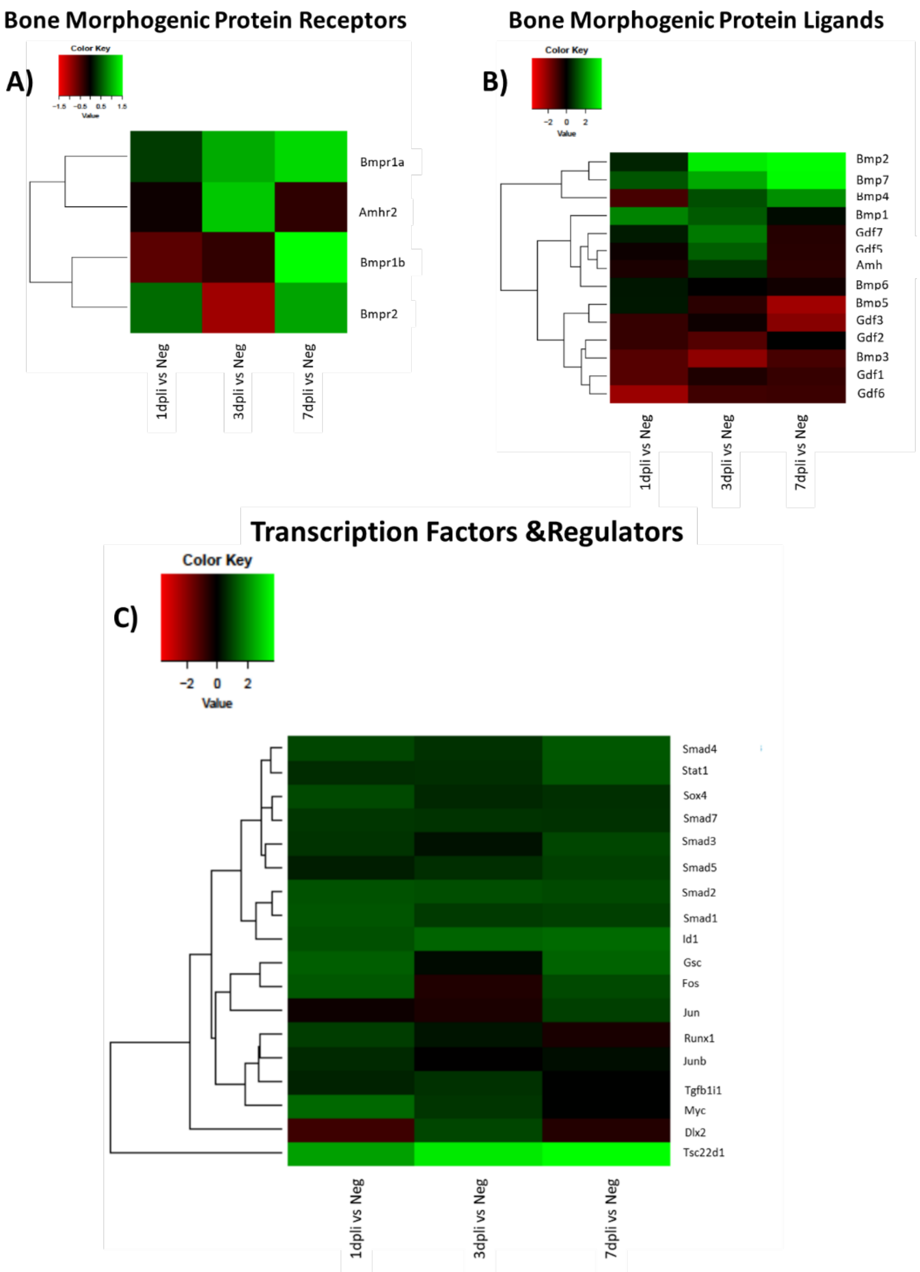

Figure S4: Heatmaps of differentially expressed genes of the TGFβ pathway in sorted proliferating and activated Müller cells in mouse. (A) Heatmap of bone morphogenetic protein receptors differentially expressed genes in sorted proliferating and activated Müller cells. (B) Heatmap of bone morphogenetic protein ligands differentially expressed genes in sorted proliferating and activated Müller cells. (C) Heatmap of transcription factors and

regulators of TGFβ signaling differentially expressed genes in sorted proliferating and activated Müller cells. Data are expressed as fold-changes compared to negative controls (uninjured retinas from age-matched, undamaged littermates).

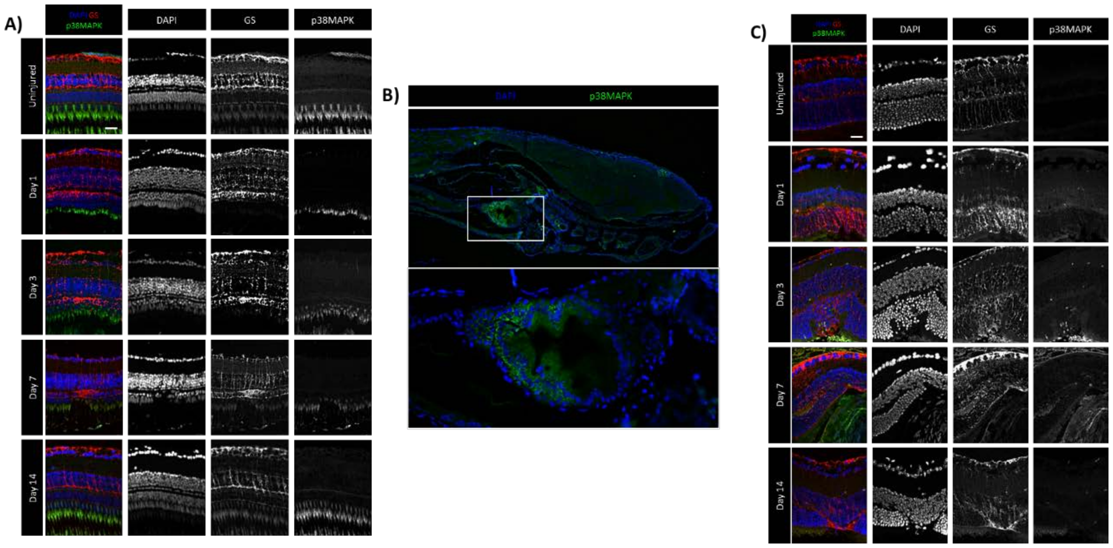

Figure S5: Absence of p38MAPK expression in Müller cells during laser injury response in zebrafish compared to mouse. (A) Analysis of Müller cells p38MAPK expression in the zebrafish retina during laser injury response and in negative controls (Uninjured). Shown are retinal sections at different time points (1, 3, 7 and 14 dpli) after laser damage induction stained for GS (red) and p38MAPK (green). (B) Shown are retinal sections of zebrafish embryo stained for p38MAPK (green). (C) Analysis of Müller cell p38MAPK expression in the murine retina during laser injury response and in negative controls (Uninjured). Shown are retinal sections at different time points (1, 3, 7 and 14 dpli) after laser damage induction stained for GS (red) and p38MAPK (green). Cell nuclei were counterstained with DAPI (blue). INL, inner nuclear layer; ONL, outer nuclear layer. Scale bar of the images equals 50 µm.

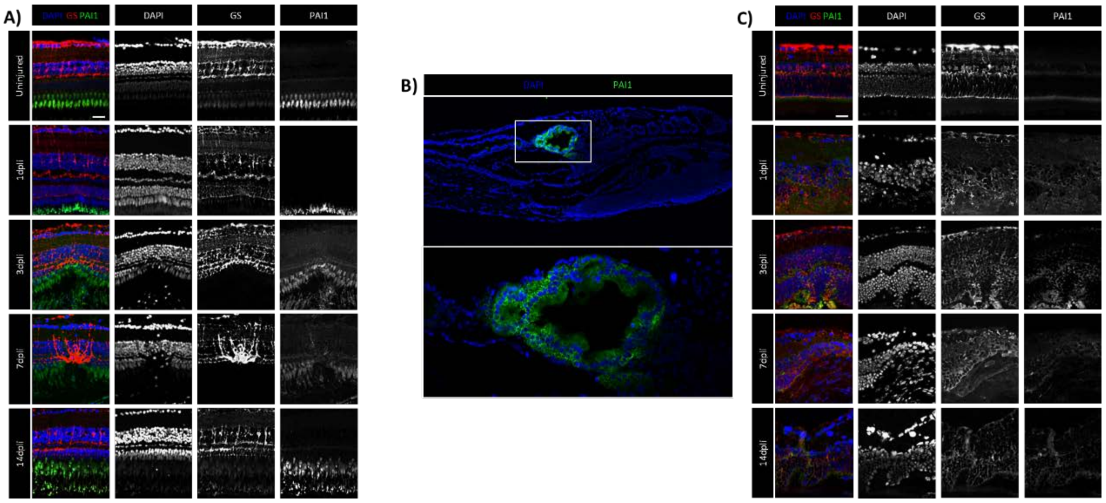

Figure S6: Absence of PAI1 expression in Müller cells during laser injury response in zebrafish compared to mouse. (A) Analysis of Müller cells PAI1 expression in the zebrafish retina during laser injury response and in negative controls (Uninjured). Shown are retinal sections at different time points (1, 3, 7 and 14 dpli) after laser damage induction stained for GS (red) and PAI1 (green). (B) Shown are retinal sections of zebrafish embryo stained for PAI1 (green). (C) Analysis of Müller cells PAI1 expression in the murine retina during laser injury response and in negative controls (Uninjured). Shown are retinal sections at different time points (1, 3, 7 and 14 dpli) after laser damage induction stained for GS (red) and PAI1 (green). Cell nuclei were counterstained with DAPI (blue). INL, inner nuclear layer; ONL, outer nuclear layer. Scale bar of the images equals 50 µm.

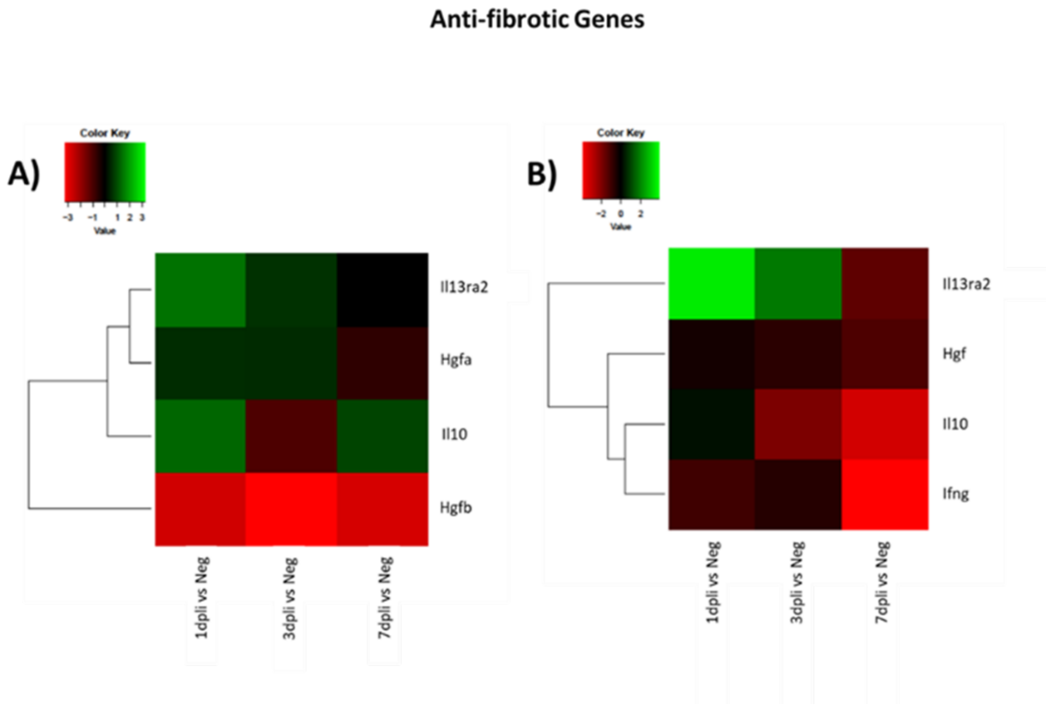

Figure S7: Comparison of transcriptional levels of anti-fibrotic genes between zebrafish and mouse in response to retinal injury. (A-B) Heatmaps of anti-fibrotic genes differentially expressed genes in sorted proliferating and activated Müller cells in zebrafish (A) and mouse (B). Data are expressed as fold-changes compared to negative controls (uninjured retinas from age-matched, undamaged animals).

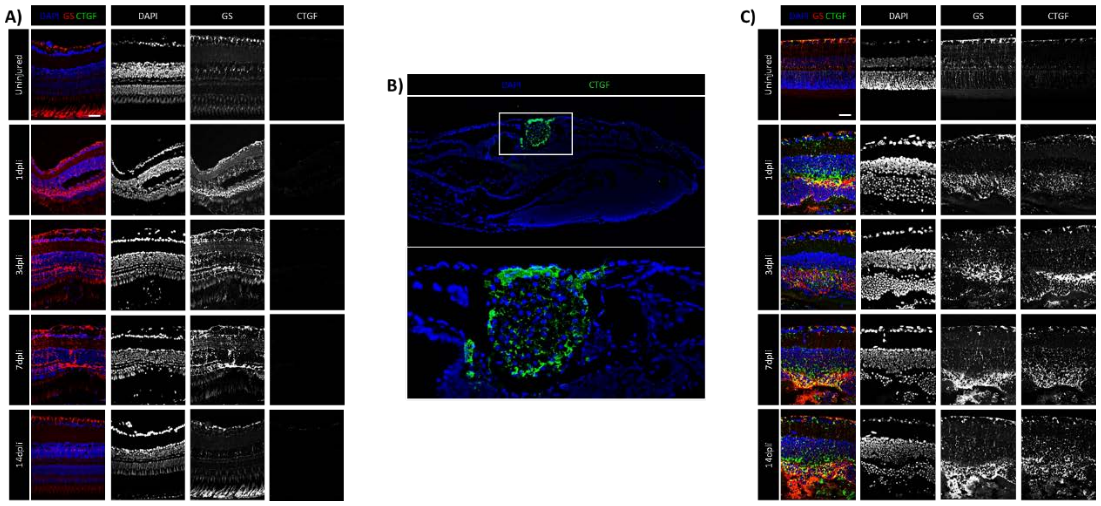

Figure S8: Absence of CTGF expression in Müller cells during laser injury response in zebrafish compared to mouse. (A) Analysis of Müller cells CTGF expression in the zebrafish retina during laser injury response and in negative controls (Uninjured). Shown are retinal sections at different time points (1, 3, 7 and 14 dpli) after laser damage induction stained for GS (red) and CTGF (green). (B) Shown are retinal sections of zebrafish embryo stained for CTGF (green). (C) Analysis of Müller cells CTGF expression in the murine retina during laser injury response and in negative controls (Uninjured). Shown are retinal sections at different time points (1, 3, 7 and 14 dpli) after laser damage induction stained for GS (red) and CTGF (green). Cell nuclei were counterstained with DAPI (blue). INL, inner nuclear layer; ONL, outer nuclear layer. Scale bar of the images equals 50 µm.

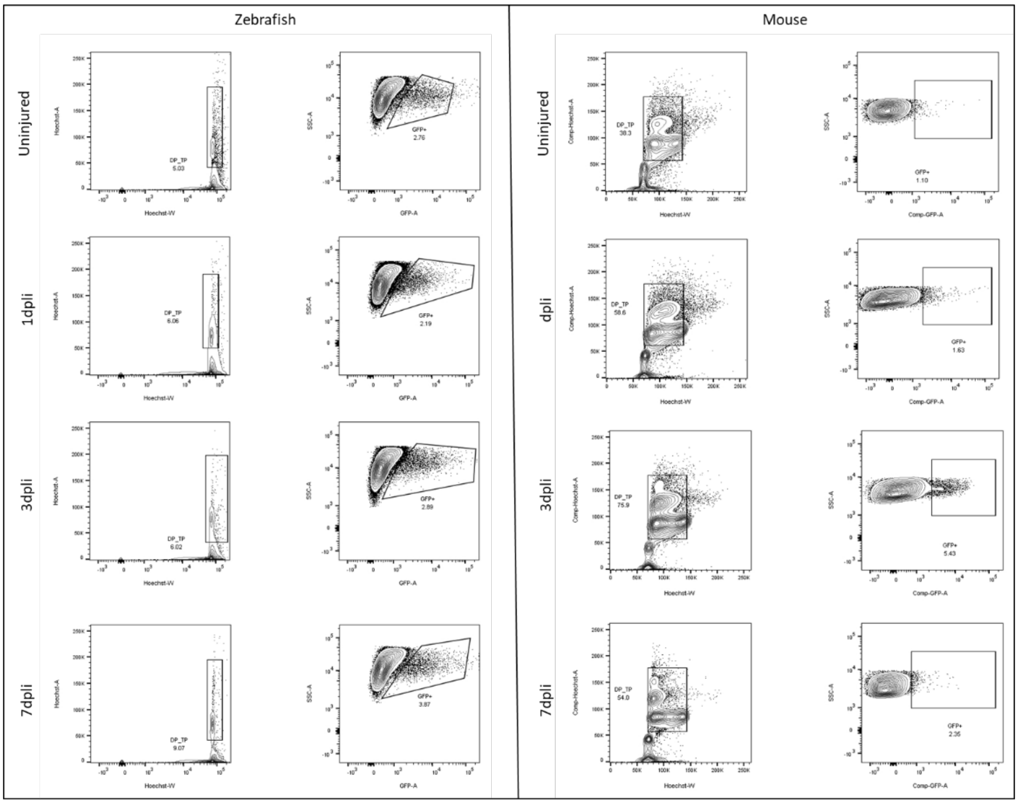

Figure S9: Cell sorting of the proliferating Müller cells after laser induction. FACS-sorted cells from gfap:gfap-GFP transgenic zebrafish (left panel) and from Rlbp1:GFP mice (right panel) stained with Hoechst 33342 Ready Flow™ Reagent, used to evaluate cell cycle distribution. Cells were sorted at different time points (1, 3 and 7 dpli) after laser damage and from negative controls (Uninjured).

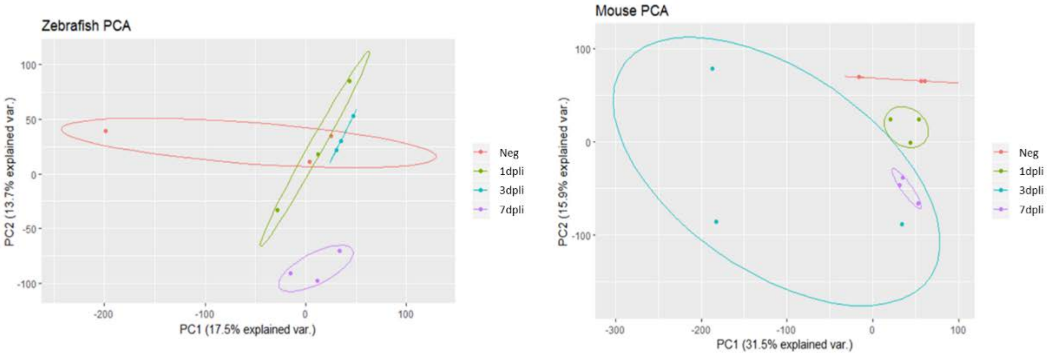

Figure S10: Principal component analysis (PCA) of RNA-seq. Samples from gfap:gfap-GFP transgenic zebrafish (left panel) and from Rlbp1:GFP mice (right panel) at different time points (1, 3 and 7 dpli) after laser injury induction and compared to negative controls (Uninjured retinas).
